# Broad and diverse roles of sphingosine-1-phosphate/sphingosine-1-phosphate receptors in the prostate

**DOI:** 10.1016/j.isci.2024.111290

**Published:** 2024-10-30

**Authors:** Daoquan Liu, Jianmin Liu, Yan Li, Lu Du, Qingqiong Cao, Liang Yang, Yongying Zhou, Ping Chen, Yuming Guo, Guang Zeng, Michael E. DiSanto, Weidong Hu, Xinhua Zhang

**Affiliations:** 1 Department of Urology, Zhongnan Hospital of Wuhan University, Wuhan 430071, China; 2 Department of Thoracic Surgery, Zhongnan Hospital of Wuhan University, Wuhan 430071, China; 3 Hubei Key Laboratory of Tumor Biological Behaviors & Hubei Provincial Clinical Research Center for Cancer, Wuhan 430071, China; 4 Department of Ultrasound, Zhongnan Hospital of Wuhan University, Wuhan 430071, China; 5 Department of Surgery and Biomedical Sciences, Cooper Medical School of Rowan University, Camden, NJ, USA

**Keywords:** natural sciences, biological sciences, biochemistry, physiology, pathophysiology

## Abstract

Benign prostatic hyperplasia (BPH) is a common condition in aging males, but its underlying pathogenesis remains unclear. Sphingosine-1-phosphate (S1P) and its receptors (S1PRs) play important roles in various diseases, while less studied in prostate. Current study attempts to clarify the expression and functional activities of S1P/S1PRs in the prostate. We discovered that S1P/S1PRs were richly expressed in the prostate, with S1PR1/2/3 localized in the epithelial/stromal compartments, while S1PR4/5 were less expressed. *In vitro*, S1P/S1PR1/S1PR3 promoted cell proliferation via AKT and ERK1/2 pathways, S1P/S1PR2/S1PR3 enhanced contraction of WPMY-1 cells and human prostate via RhoA/ROCK pathway, while S1P/S1PR1/S1PR2/S1PR3 alleviated the inflammation response via STAT3 pathway. *In vivo*, S1P and S1PR1/3 agonists (SEW2871, CYM5541) led to prostate enlargement in rats, while S1PR1/3 antagonists (W-146, TY-52156) suppressed testosterone-induced BPH. Overall, this study suggests that S1P/S1PRs play a critical role in the development of BPH and may be a promising therapeutic target for BPH treatment.

## Highlights

• S1PR1/2/3 are richly expressed in the prostate, localized in the epithelial and stromal parts
• S1P/S1PR1/S1PR3 promotes cell proliferation via AKT and ERK1/2 pathways
• S1P/S1PR2/S1PR3 enhances contraction of WPMY-1 cells and human prostate via RhoA/ROCK pathway
• S1P/S1PR1/S1PR2/S1PR3 alleviates the inflammation response via STAT3 pathway

## Graphical abstract

**Figure:**
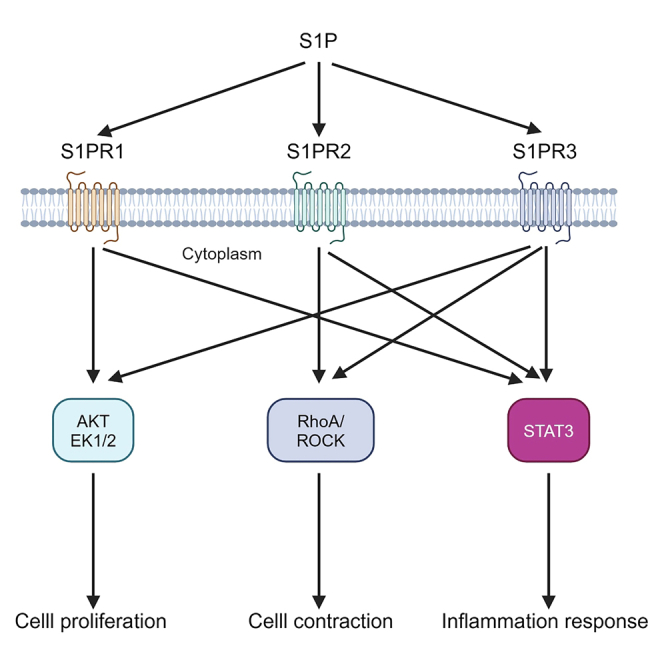

## Introduction

Benign prostatic hyperplasia (BPH) is one of the most common conditions in elderly men and leads to lower urinary tract symptoms (LUTS) and erectile dysfunction (ED),^1^ both of which severely impact the patients’ quality of life. The incidence rate of BPH is age-dependent and reaches 50% in men over the age of 60, but rapidly increases to 90% for men in their eighth decade of life or older.^1^ With the progressive aging of the general population, BPH has emerged as a huge medical and socioeconomic problem worldwide.

BPH is caused by increased prostate volume (static factor) and increased prostate tension (active and passive tension).^1^ Aging and androgens are the two major pathogenic factors.^2^ Increased estrogen/androgen ratio, imbalance of apoptosis and proliferation, stromal-epithelial interactions, growth factors and cytokines, inflammation, and genetic and familial factors have been demonstrated to be involved in the development of BPH.^1^ However, the exact etiology and pathogenesis of BPH have not been fully elucidated. Although current first-line pharmacotherapy, such as alpha-blockers, 5-alpha reductase inhibitors and PDE5 inhibitors can be effective, there are some bothersome side effects.^1^ More importantly, these drugs could not completely prevent the progression of BPH and ultimately 20% of BPH patients still require surgical procedures.^1^ Hence, more deeply probing studies of BPH, especially exploring the molecular mechanism and rediscovering therapeutic targets, are essential.

Sphingosine-1-phosphate (S1P)/Sphingosine-1-phosphate receptors (S1PRs) has been the focus of intensive investigations for nearly three decades.^3^ S1P, a bioactive sphingolipid metabolite, is produced by the phosphorylation of sphingosine via sphingosine kinases 1/2 (SphK1/2).^3^^,^^4^^,^^5^^,^^6^ Currently, two different SphK (SphK1, SphK2) isoforms have been found in mammals.^6^^,^^7^ SphK1 is predominantly localized in the cytoplasm and cytomembrane, with a pro-survival effect, while SphK2 is mainly localized in the mitochondria, cell nucleus, and endoplasmic reticulum, with roles in inducing apoptosis.^5^^,^^8^ S1P has been demonstrated to act both as a second messenger and as a ligand of G-protein-coupled S1P receptors (S1PRs),^5^^,^^8^ and to be involved in a wide array of biological processes such as cell proliferation, survival, migration, inflammation, vasorelaxation, vasocontraction, and others.^3^^,^^7^^,^^9^^,^^10^^,^^11^ A number of biological processes are initiated by S1P binding and activating S1PRs.^5^ To date, five S1PRs have been identified and designated S1PR1-5. S1PR1/2/3 are found in all tissues of mammals, while S1PR4 only in lymphoid tissues and lung, and S1PR5 just in brain and skin.^5^^,^^12^

S1P was originally found to promote proliferation of fibroblasts by *Olivera* et al. in 1993, but their study failed to demonstrate which receptor its bound to.^3^ Since then, dozens of studies were performed to explore the effects of various S1PRs on cell proliferation. *Chen* et al. revealed that the SphK1/S1P/S1PR1/S1PR3 cascade was essential for human amniotic cells proliferation^13^; *Wang* et al. discovered that S1P enhanced growth of endothelial progenitor cells via S1PR1/S1PR3.^14^ Of note, the effect of S1P/S1PRs on cancer cell growth has been widely investigated, including in prostate cancer. *Brizuela* et al. indicated that osteoblast-derived S1P could induce proliferation of prostate cancer cells by binding to S1PR1.^15^ In general, the majority of studies have shown that cell proliferation is associated with S1PR1 and S1PR3.^13^^,^^14^^,^^16^^,^^17^^,^^18^ In addition, a few studies elucidated the functions of S1PR2 in the growth of the smooth muscle (SM) cell, epithelial stem cell, and cholangiocyte.^18^^,^^19^^,^^20^^,^^21^

Also, S1P was shown to be involved in the immune response and inflammation. S1PRs are widely expressed in various immune cells.^5^^,^^9^^,^^22^ It has been clarified that there is an S1P concentration gradient between lymphoid organs and circulation, which is important in transporting lymphocytes via S1PR1.^5^^,^^8^^,^^23^ Fingolimod, as a nonselective S1PR agonist (except for only weak binding to S1PR2), has been used for the treatment of relapsing-remitting multiple sclerosis based on this gradient.^9^^,^^24^^,^^25^ Siponimod has higher selectivity and fewer side effects, and was approved by the Food and Drug Administration (FDA) to treat secondary progressive multiple sclerosis in 2019.^12^^,^^24^ These agents induce the homing of lymphocytes through the functional inhibition of S1PR1 and thus play an immunosuppressive role.^5^^,^^9^^,^^12^^,^^23^^,^^24^ Moreover, *Zhang* et al. revealed that FTY720, a modulator of S1PRs, could suppress rat experimental autoimmune prostatitis.^26^ Additionally, S1P concentration gradients mediate the migration of immune cells to the site of inflammation via S1PR1.^22^ On the other hand, the S1P/S1PRs axis plays an important role in the maintenance of vascular integrity and strengthens the adherens junction to limit exaggerated inflammation.^9^^,^^27^ A previous study revealed that S1P/S1PRs were associated with colitis, inflammatory bowel diseases, and inflammation-associated colon cancer.^22^

S1P, as a latest addition to the family of vasoactive substances, has essential roles in vasorelaxation and vasocontraction. Many studies demonstrated that S1P-mediated vasodilation by binding to S1PR1/3 via eNOS signaling pathway in endothelial cells,^10^^,^^28^ while inducing SM contraction by activating S1PR2/3 via RhoA/ROCK signaling pathway in SM cells.^10^^,^^11^^,^^28^ In addition to vascular SM, S1P also induces contraction of SM cells in the bladder, uterus, gastrointestinal tract, bronchus, and coronary artery.^10^^,^^11^^,^^29^^,^^30^

Of note, S1P/S1PRs modulate a wide range of biological functions, which include cell proliferation, cell survival, and SM tone, and participate in the initiation and development of multiple diseases. However, for a specific tissue, the biological function of the S1P signaling pathway depends upon the species, the expression and localization of S1PR subtypes, and the physio-pathological state of the organism. With regard to prostate, the role of S1P/S1PRs has never been determined. The aim of current study was to determine expression and functional activities of S1P/S1PRs in the prostate. Firstly, we investigated the expression of S1P/S1PRs in the prostate. Then we added exogenous S1P, silenced and overexpressed S1PRs in cultured human epithelial and stromal cells and examined the alterations of cell proliferation, cell cycle, inflammation, and cell contraction, as well as associated signaling pathways. Finally, we injected the S1P and agonists of S1PRs into the ventral prostate of rats to explore the *in vivo* effect of S1P/S1PRs.

## Results

### The concentration of S1P in human normal prostate tissues

The average concentration of S1P was determined to be 26.86 ± 7.29 pmol/mg using ELISA in 10 human normal prostates.

The expression and localization of S1PRs in various rat and human tissues, as well as human prostate cell lines.

**Figure 1:**
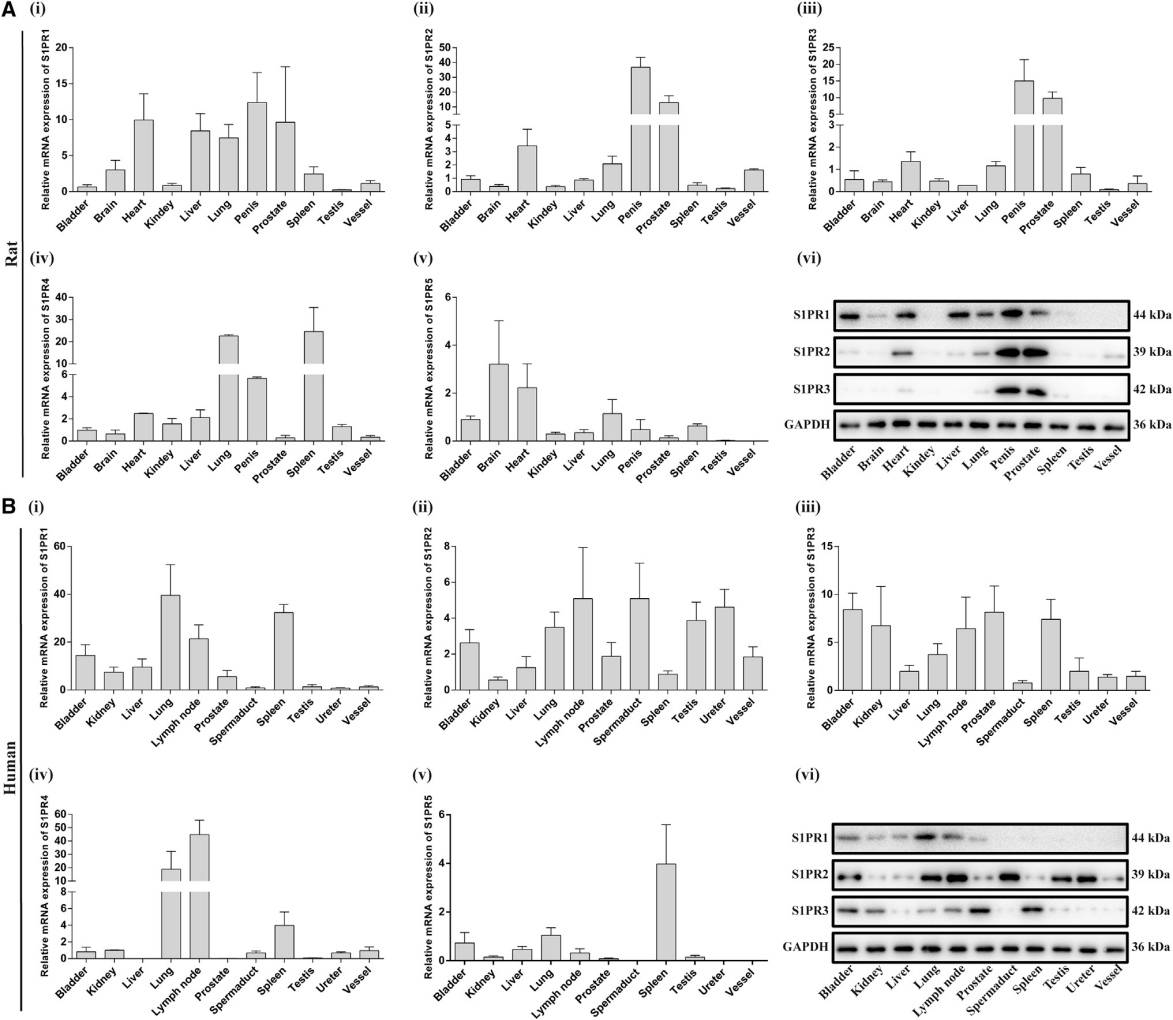
The expression of S1PRs in various human and rat tissues (A) The mRNA and protein levels of S1PRs in various rat tissues (*n* = 10). (B) The mRNA and protein levels of S1PRs in various human tissues (*n* = 10). Data are represented as M ± SD.

**Figure 2:**
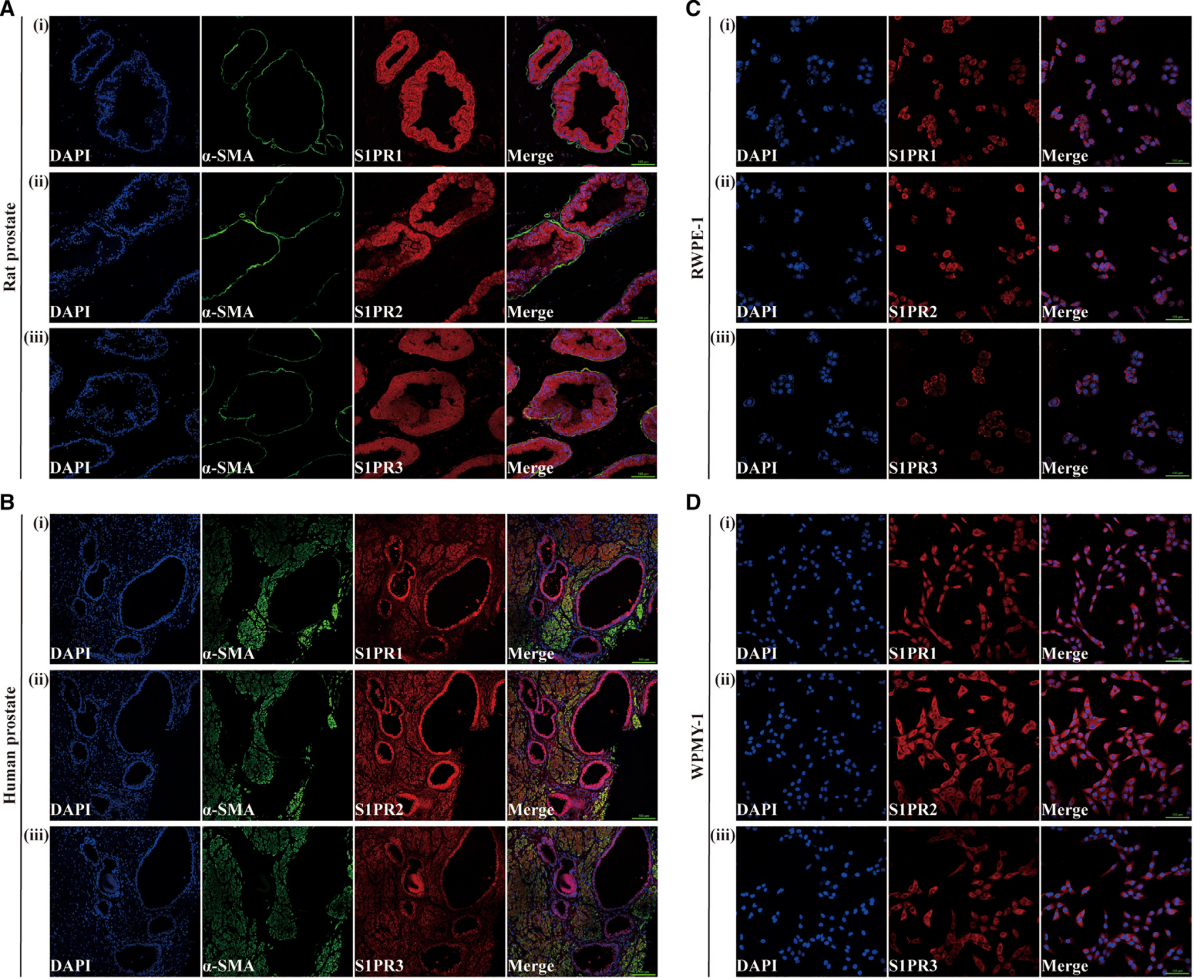
The expression and localization of S1PR1/2/3 in rat/human prostate tissues and human prostate cell lines (A and B) Immunofluorescence localization of S1PR1/2/3 in normal rat/human prostate. Cy3-immunofluorescence (red) indicates S1PR1/2/3 protein staining, Cy3-immunofluorescence (green) indicates α-SMA protein staining, and DAPI (blue) indicates nuclear staining. (C and D) Immunofluorescence localization of S1PR1/2/3 in RWPE-1 and WPMY-1 cells. DAPI (blue) indicates nuclear staining and Cy3-immunofluorescence (red) indicates S1PR1/2/3 protein staining. Representative micrographs are shown. All scale bars are 100 μm.

We investigated the mRNA expression levels of S1PRs in various rat and human tissues (Tables 1 and 2). For rat tissues, S1PR1 exhibited high expression in the heart, liver, lungs, penis, and prostate. S1PR2 showed high expression in the heart, lungs, penis, prostate, and vessel. S1PR3 demonstrated high expression in the heart, lungs, penis, and prostate. S1PR4 was predominantly expressed in the lungs, penis, and spleen, while S1PR5 showed high expression in the brain, heart, and lungs (Figure 1A). For human tissues, S1PR1 was highly expressed in the bladder, kidney, liver, lungs, lymph node, prostate, and spleen. S1PR2 was highly expressed in the bladder, liver, lungs, lymph node, prostate, spermduct, testis, ureter, and vessel. S1PR3 was highly expressed in the bladder, kidney, lungs, lymph node, prostate, and spleen. S1PR4 was highly expressed in the lungs, lymph node, and spleen. S1PR5 was highly expressed in the bladder, lungs, and spleen (Figure 1B). In comparison to other tissues, S1PR1/2/3 were highly expressed but S1PR4/5 were nearly undetectable in both rat and human prostate (Figure 1). Consequently, we proceeded to examine the protein levels of S1PR1/2/3, which were also richly expressed in both rat and human prostate (Figure 1, Tables 3, and 4). Moreover, tissue immunofluorescent staining demonstrated that S1PR1/2/3 were localized both in epithelia and stroma of rat and human prostate (Figures 2A and 2B). In addition, cell immunofluorescent staining indicated that S1PR1/2/3 were both expressed in cultured human RWPE-1 and WPMY-1 cells (Figures 2C and 2D).

**Table 1:** Primer sequence used for qPCR in human tissues

Target gene		Human (5′ to 3′)
S1PR1	Forward	GTGTTCAGTCTCCTCGCCAT
	Reverse	GCAGGAAGAGGCGGAAGTTA
S1PR2	Forward	CATCCTCTGTTGCGCCATTG
	Reverse	CATTGCCGAGTGGAACTTGC
S1PR3	Forward	TGGTCCCCACTCTTCATCCT
	Reverse	CAGCCAACACGATGAACCAC
S1PR4	Forward	GACGCTGGGTCTACTATTGCC
	Reverse	CCTCCCGTAGGAACCACTG
S1PR5	Forward	TAGCCGTGTTGTTGGTGCTC
	Reverse	CAGCAGATCCGACAACGTGA
IL-6	Forward	GAACTCCTTCTCCACAAGCG
	Reverse	GCCTCTTTGCTGCTTTCACA
IL-8	Forward	TCCAAACCTTTCCACCCCAAA
	Reverse	TTTCTGTGTTGGCGCAGTGT
TNF-α	Forward	GGTCCTCTTCAAGGGCCAAG
	Reverse	TCACAGGGCAATGATCCCAA
GAPDH	Forward	ATCCCATCACCATCTTCCAGGAG
	Reverse	CCTGCTTCACCACCTTCTTGATG

**Table 2:** Primer sequence used for qPCR in rat tissues

Target gene		Human (5′ to 3′)
S1PR1	Forward	CCAGTGGTTAAGGCTCTCCG
	Reverse	TGGTCCTTCTCCACTCCGAT
S1PR2	Forward	AGCCTTCATCACGCTCTCTG
	Reverse	GCCACCCAGAATCAGCGATA
S1PR3	Forward	ATCACCACCATCCTCTTCTTGG
	Reverse	CCACACTGTTGGAGACAGGC
S1PR4	Forward	ATCTATTCCTTCCGCAGCCG
	Reverse	AGTCCGAAAACTGTCCCTGG
S1PR5	Forward	TATGTGCTCTTCTGCGTGCT
	Reverse	GGCACGCGACATCCAGTAAT
IL-6	Forward	TCCTACCCCAACTTCCAATGC
	Reverse	GGTCTTGGTCCTTAGCCACT
IL-8	Forward	CCGAAGTCATAGCCACACTCA
	Reverse	GGGGACACCCTTTAGCATCTT
TNF-α	Forward	ATCGGTCCCAACAAGGAGGA
	Reverse	TCCGCTTGGTGGTTTGCTAC
β-actin	Forward	ACCAACTGGGACGATATGGAGAAGA
	Reverse	TACGACCAGAGGCATACAGGGACAA

**Table 3:** List of primary antibodies

Antigens	Species	Dilution (IF)	Dilution (WB)	Supplier
CDK6	Rabbit, monoclonal	–	1:1000	CST, Cat. 13331
CDK4	Rabbit, monoclonal	–	1:1000	CST, Cat. 12790
Cyclin D1	Rabbit, monoclonal	–	1:1000	CST, Cat. 2978
TNF-α	Rabbit, polyclonal	–	1:1000	Abclonal, Cat. A0277
IL-6	Rabbit, polyclonal	–	1:1000	Abclonal, Cat. A0286
IL-8	Rabbit, polyclonal	–	1:1000	Abclonal, Cat. A2541
S1PR1	Rabbit, polyclonal	1:200	1:1000	Abcam, Cat. ab11424
S1PR2	Rabbit, polyclonal	1:200	1:1000	Abcam, Cat. ab235919
S1PR3	Rabbit, monoclonal	1:100	1:1000	Abcam, Cat. ab126622
pAKT	Rabbit, monoclonal	–	1:1000	Abclonal, Cat. AP0637
AKT	Rabbit, monoclonal	–	1:1000	Abclonal, Cat. A17909
pERK1/2	Rabbit, monoclonal	–	1:1000	Abclonal, Cat. AP0472
ERK1/2	Rabbit, monoclonal	–	1:1000	Abclonal, Cat. A4782
pSTAT3	Rabbit, monoclonal	–	1:1000	Abclonal, Cat. AP0715
STAT3	Rabbit, polyclonal	–	1:1000	Abclonal, Cat. A1192
RhoA	Rabbit, monoclonal	–	1:1000	Abclonal, Cat. A19106
ROCK1	Rabbit, monoclonal	–	1:1000	Abclonal, Cat. A11158
ROCK2	Rabbit, monoclonal	–	1:1000	Abclonal, Cat. A2395
GAPDH	Rabbit, monoclonal	–	1:1000	Abclonal, Cat. AC001

**Table 4:** List of secondary antibodies used for western blot

Secondary Detection System Used	Host	Dilution used	Supplier
Anti-Rabbit-IgG (H + L)-HRP	Goat	1:10000 (WB)	Sungene Biotech, Cat. #LK2001
Anti-Rabbit IgG (H + L), F(ab')2 fragment (Alexa Fluor® 488 Conjugate)	Goat	1:50 (IF)	Cell Signaling Technology, USA, cat. no. 4412
Hoechst 33342 (1 mg/mL) nucleic acid staining (DAPI)	–	1:750 (IF)	Molecular Probes/Invitrogen, Carlsbad, CA, USA, cat. no. A11007

### S1P/S1PR1/S1PR3 promoted proliferation of prostate cells via ERK1/2 and AKT pathways with no effect on apoptosis

**Figure 3:**
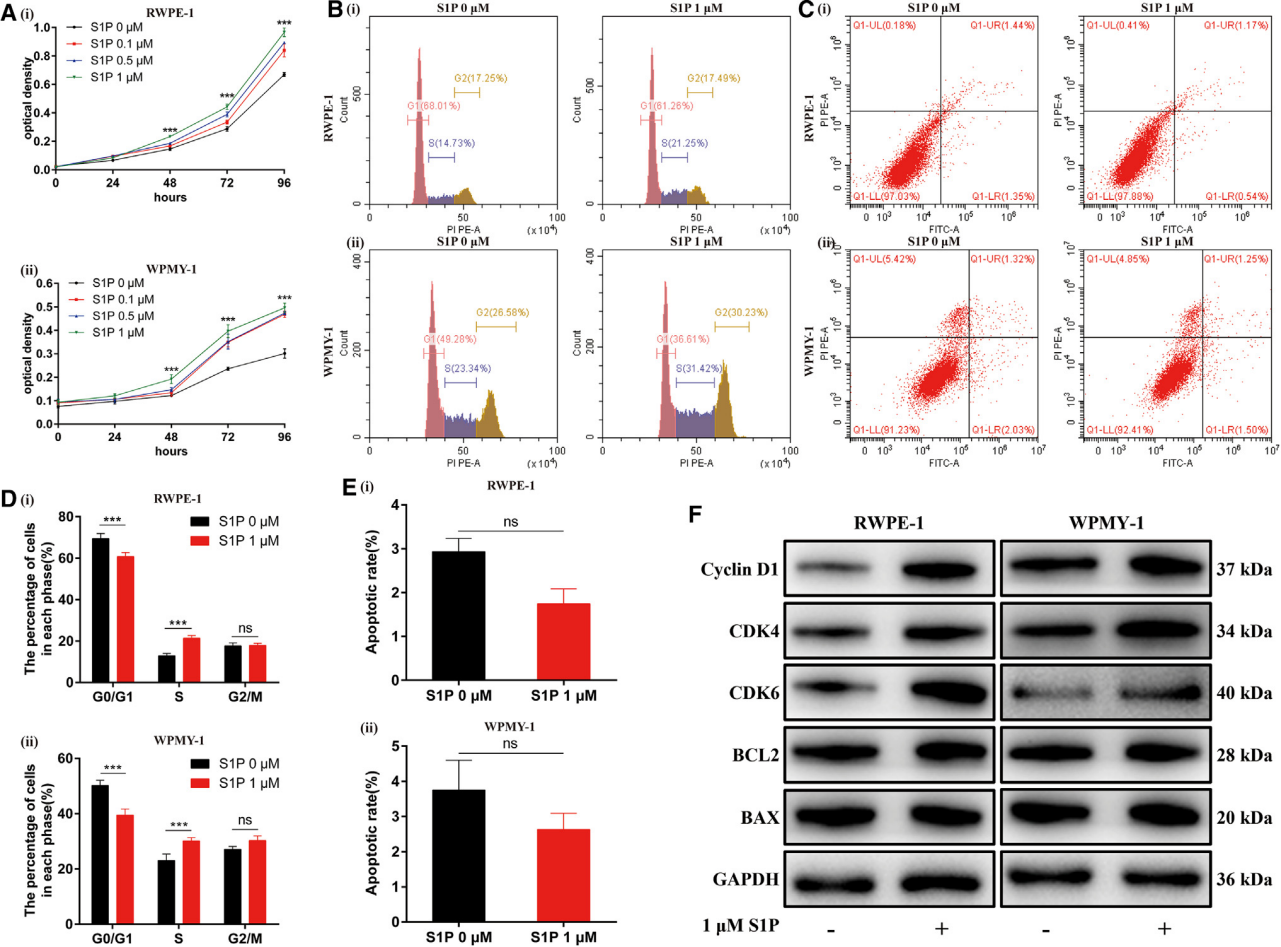
S1P promoted proliferation of prostate cells but had no effect on apoptosis (A) The effect of S1P on cell proliferation. (B) The effect of S1P on cell cycle. (C) The effect of S1P on cell apoptosis. (D) Statistical analysis of percentages (%) of cells at each stage of cell cycle. (E) Statistical analysis of apoptotic rate (%). (F) Western blot assay of cell cycle- and cell apoptosis-related proteins. Data are represented as M ± SD. ∗∗∗*p* < 0.001 and ns means no significant difference.

We firstly treated cultured human prostate cells with a gradient concentration of S1P (0, 0.1, 0.5, 1 μM) and found that S1P promoted cell proliferation in a concentration-dependent manner (Figure 3A). Due to the best effect, 1 μM was used for subsequent experiments. Flow cytometry analysis demonstrated that S1P significantly enhanced the transition from G0/G1 phase to S phase with no effect on cell apoptosis (Figures 3B–3E). Consistently, expression of cell cycle-associated markers (Cyclin D1, CDK4, CDK6) was significantly upregulated at the protein level, while expression of cell apoptosis-associated markers (BAX, BCL2) showed no alteration (Figure 3F). In short, S1P indeed promoted proliferation of prostate cells.

**Figure 4:**
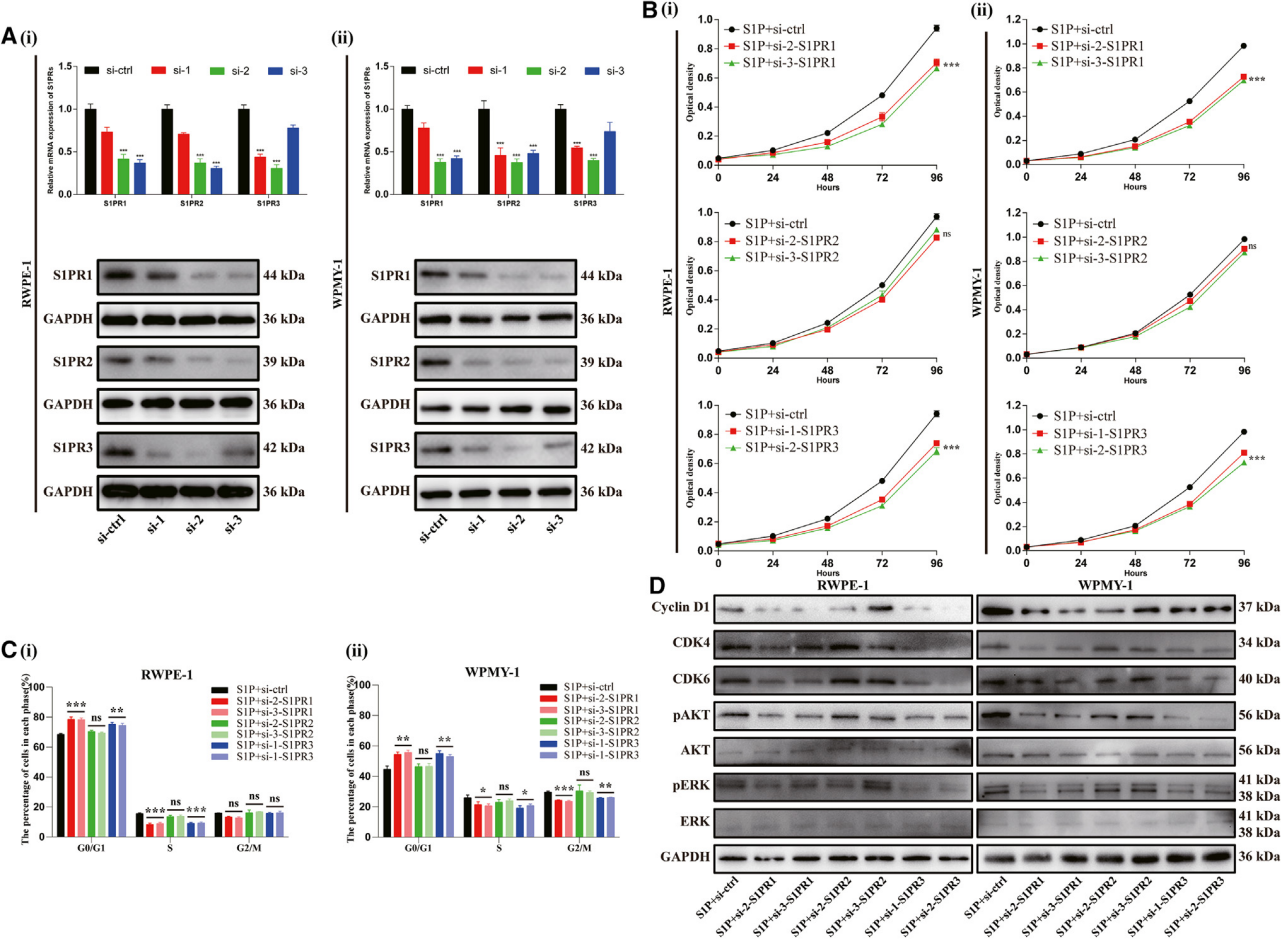
Knockdown of S1PR1/3 inhibited S1P-induced proliferation of RWPE-1 and WPMY-1 cells via inhibition of AKT and ERK1/2 pathway (A) Knockdown efficiency of S1PR1/2/3 at mRNA and protein levels. (B) The effect of S1PRs knockdown on cell proliferation. (C) Statistical analysis of percentages (%) of cells at each stage of cell cycle. (D) Western blot assay of cell cycle- and pathway-related proteins. Data are represented as M ± SD. ∗*p* < 0.05, ∗∗*p* < 0.01, ∗∗∗*p* < 0.001 and ns means no significant difference.

We further explored which S1PR played essential roles in S1P-induced cell proliferation through S1PR1/2/3 knockdown studies. As shown in Figure 4A, Tables 1, and 5, knockdown efficiency of siRNAs was verified by RT-qPCR and western blot. For each receptor, two siRNAs with highest knockdown efficiency were used for subsequent experiments. Knockdown of S1PR1 and S1PR3 significantly inhibited S1P-induced proliferation of both cell lines (Figure 4B) with an increase of G0/G1 phase cells and a decrease of S phase cells (Figures 4C and S1). In WPMY-1 cells, a decrease of G2/M phase cells was also identified (Figures 4C and S1). However, silence of S1PR1 and S1PR3 exhibited no effect on cell apoptosis (Figure S2). Moreover, the expression of proteins involved in G0/G1 phase (Cyclin D1, CDK4, CDK6) and potential associated signaling pathway (pAKT, pERK1/2) were significantly attenuated when S1PR1 and S1PR3 silenced (Figure 4D). Interestingly, knockdown of S1PR2 had no significant effect on aforementioned biological process (Figure 4; Figures S1 and S2).

**Table 5:** Sense sequences of siRNA

		sense sequences (5′ to 3′)
si-con	Forward	UUCUCCGAACGUGUCACGUTT
	Reverse	ACGUGACACGUUCGGAGAATT
si-1-S1PR1	Forward	CCGCAGCAAAUCGGACAAUTT
	Reverse	AUUGUCCGAUUUGCUGCGGTT
si-2-S1PR1	Forward	CCAGAGACCAUUAUGUCUUTT
	Reverse	AAGACAUAAUGGUCUCUGGTT
si-3-S1PR1	Forward	UCUGGAAACGUCAACUCUUTT
	Reverse	AAGAGUUGACGUUUCCAGATT
si-1-S1PR2	Forward	GCUUGUACUCGGAGUACCUTT
	Reverse	AGGUACUCCGAGUACAAGCTT
si-2-S1PR2	Forward	CCUUCGUAGCCAAUACCUUTT
	Reverse	AAGGUAUUGGCUACGAAGGTT
si-3-S1PR2	Forward	CCUCUCUACGCCAAGCAUUTT
	Reverse	AAUGCUUGGCGUAGAGAGGTT
si-1-S1PR3	Forward	GCAUCGCUUACAAGGUCAATT
	Reverse	UUGACCUUGUAAGCGAUGCTT
si-2-S1PR3	Forward	CGGCACUUGACAAUGAUCATT
	Reverse	UGAUCAUUGUCAAGUGCCGTT
si-3-S1PR3	Forward	GCACUUCAGAAUGGGAUCUTT
	Reverse	AGAUCCCAUUCUGAAGUGCTT

**Figure 5:**
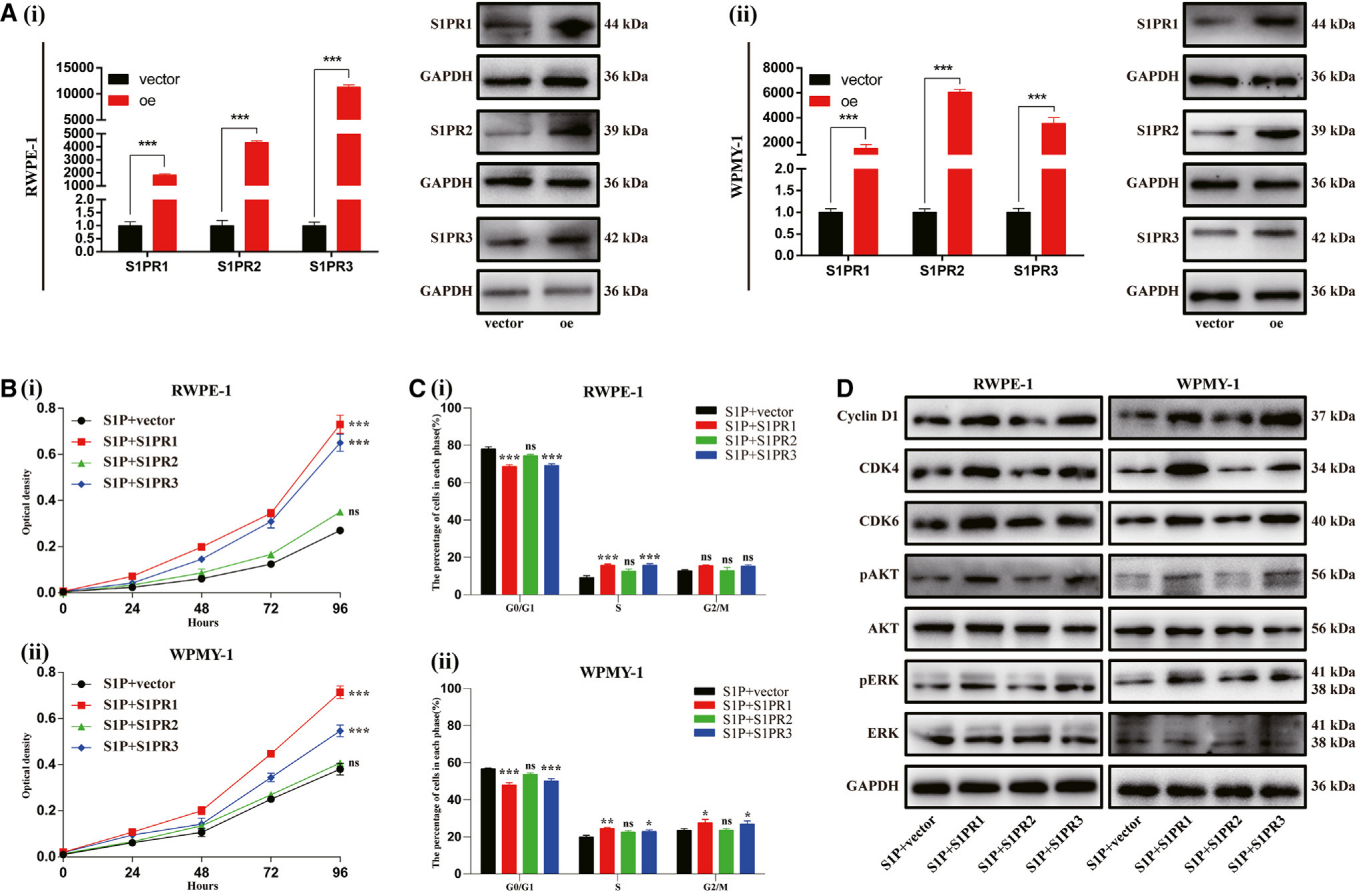
Overexpression of S1PR1/3 promoted S1P-induced proliferation of RWPE-1 and WPMY-1 cells via activation of AKT and ERK1/2 pathway (A) Overexpression efficiency of S1PR1/2/3 at mRNA and protein levels. (B) The effect of S1PRs overexpression on cell proliferation. (C) Statistical analysis of percentages (%) of cells at each stage of cell cycle. (D) Western blot assay of cell cycle- and pathway-related proteins. Data are represented as M ± SD. ∗*p* < 0.05, ∗∗*p* < 0.01, ∗∗∗*p* < 0.001 and ns means no significant difference.

We also overexpressed S1PR1/2/3 via plasmids in both cell lines. The overexpression efficiency was validated with RT-qPCR and western blot (Figure 5A). Cell proliferation and the transition from G0/G1 phase to S phase of RWPE-1 and WPMY-1 cells were enhanced when S1PR1 and S1PR3 overexpressed (Figures 5B and 5C; Figure S3A). Similar to knockdown experiments, overexpression of S1PR2 showed no significant effect on this biological process (Figures 5B and 5C; Figure S3A). Additionally, overexpression of S1PR1 and S1PR3 increased the expression of Cyclin D1, CDK4, and CDK6 and amplified the phosphorylation of AKT and ERK1/2 (Figure 5D). Again, the rates of apoptotic cells were not significantly changed no matter which S1PR was overexpressed (Figure S3B).

**Figure 6:**
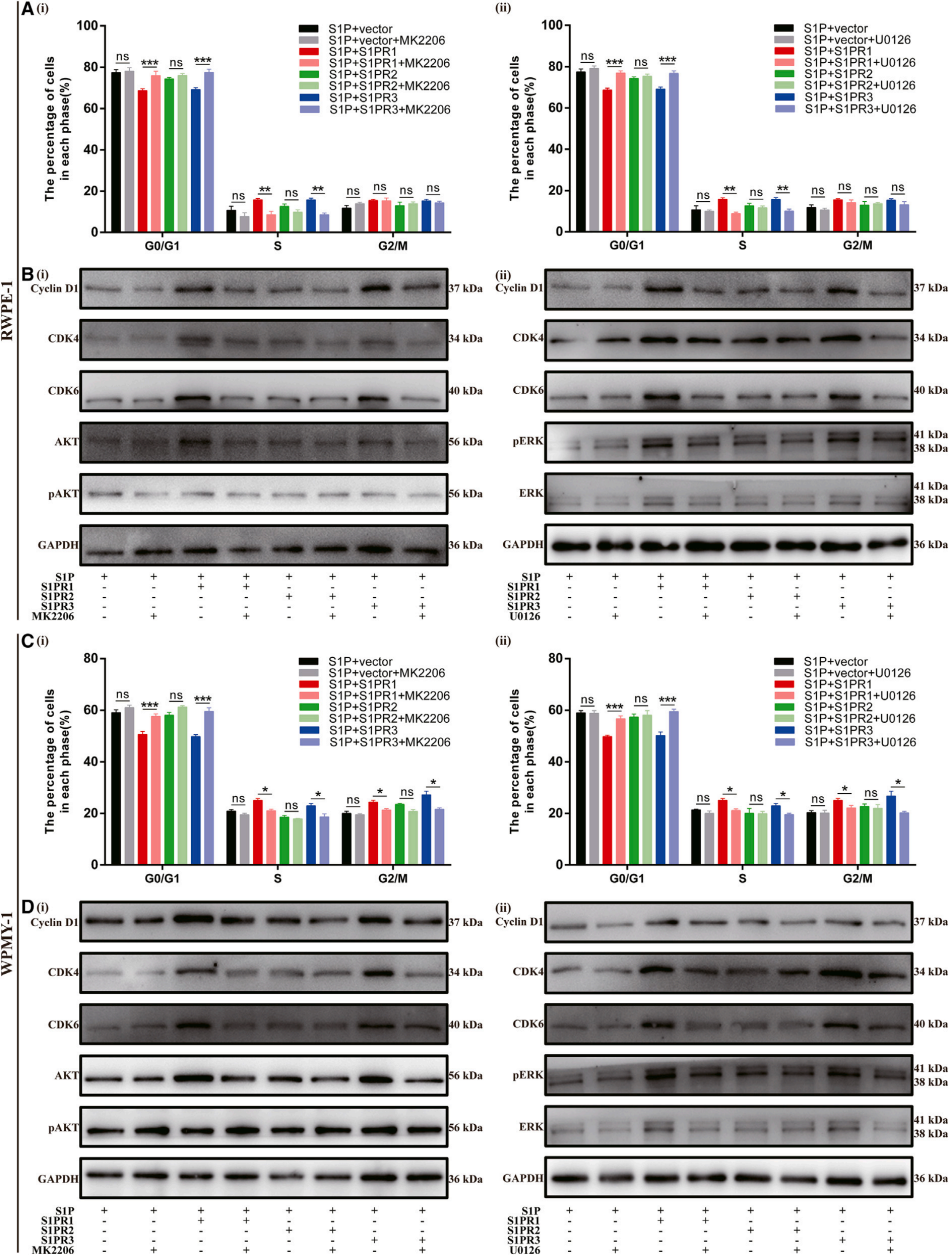
MK2206 and U0126 rescued the alterations induced by S1P and S1PR1/3 overexpression (A and C) Statistical analysis of percentages (%) of cells at each stage. (B and D) Western blot assay of cell cycle- and pathway-related proteins. Data are represented as M ± SD. ∗*p* < 0.05, ∗∗*p* < 0.01, ∗∗∗*p* < 0.001 and ns means no significant difference.

To validate if S1P/S1PRs played essential roles in cell proliferation through AKT and ERK1/2 pathway, we further incubated MK2206 (inhibitor of AKT pathway) and U0126 (inhibitor of ERK1/2 pathway) with cells transfected with plasmids, respectively. Exogenous addition of MK2206 and U0126, which rescued the alterations induced by S1P and S1PR1/3 overexpression, caused cell-cycle arrest at G0/G1 phase and decreased the expression of proteins involved in G0/G1 phase (Cyclin D1, CDK4, CDK6), along with the attenuated phosphorylation of AKT and ERK1/2 (Figure 6; Figures S4 and S5). Additionally, exogenous addition of MK2206 and U0126 had no effect on cell apoptosis (Figures S6 and S7). Thus, it indicated that S1P/S1PR1/S1PR3 did induce cell proliferation via AKT and ERK1/2 pathway.

### S1P/S1PR2/S1PR3 induced contraction of WPMY-1 cells and potentiated phenylephrine (PE)-mediated contraction of human prostate strips via RhoA/ROCK pathway

**Figure 7:**
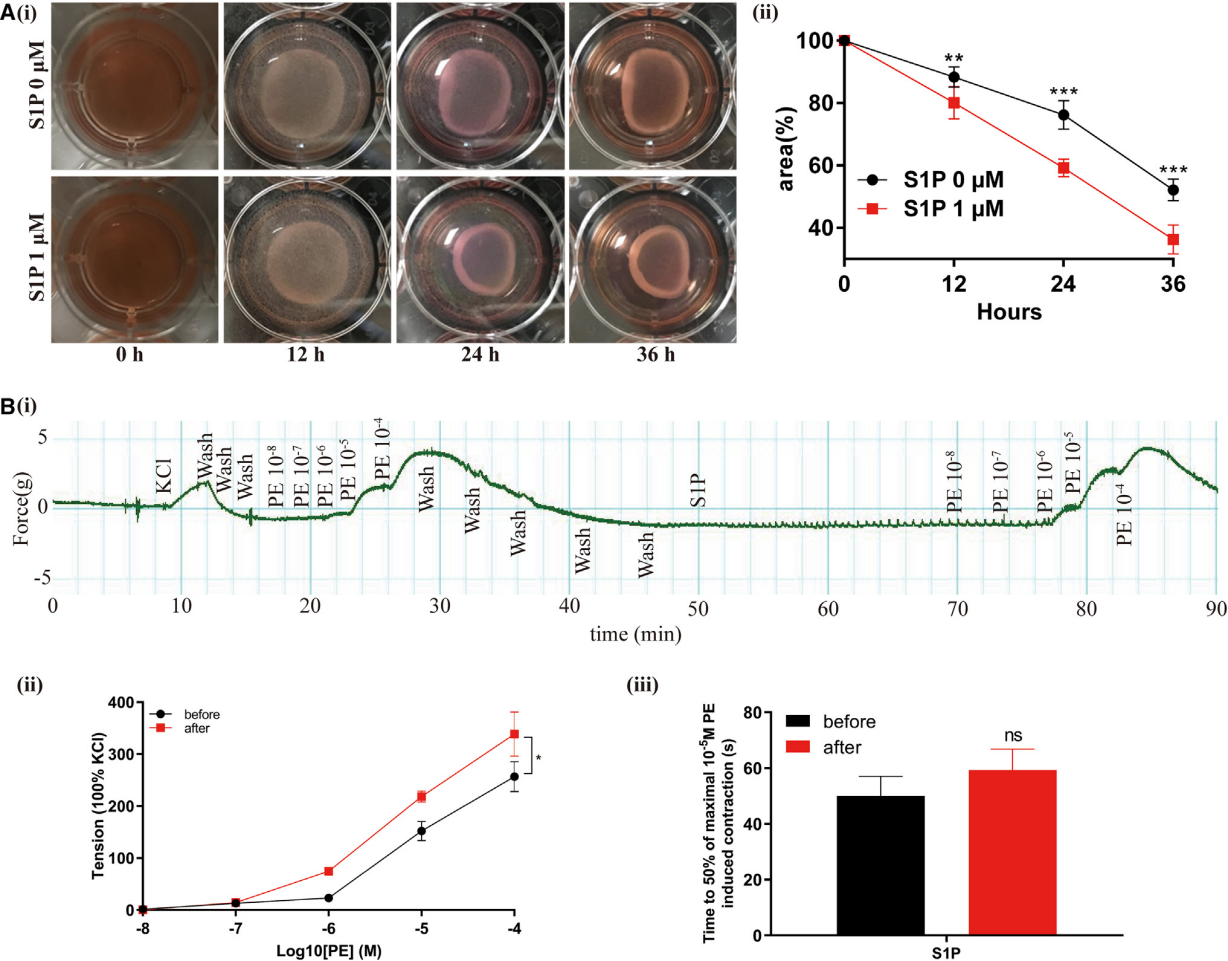
S1P induced cell contraction and potentiated PE mediated contraction of human prostate strips (A) (i) The effect of S1P on contraction of WPMY-1 cells, (ii) Statistical analysis of the percentages of the area of collagen gels. (B) (i) The effect of S1P on contraction of human prostate tissue strips, (ii) Statistical analysis of tension (KCl = 100%), (iii) Statistical analysis of time to 50% maximal 10^−5^ M PE induced contraction. Data are represented as M ± SD. ∗*p* < 0.05, ∗∗*p* < 0.01, ∗∗∗*p* < 0.001 and ns means no significant difference.

We performed collagen-based cell contraction assay to investigate the effect of S1P on contraction of WPMY-1 cells. As demonstrated in Figure 7A, S1P induced a stronger decrease of the area of collagen gels after 12 h treatment. We further examined the involvement of S1P in the contraction of human normal prostate tissues via *in vitro* organ bath studies. Although S1P cannot induce significant contraction at any doses (data not shown), it could enhance the response of human prostate tissue to gradient concentration PE (10^−8^-10^−4^ M). As shown in Figure 7B, the tension produced by gradient concentration of PE after incubation with S1P was significantly higher than that without S1P. However, time to reach 50% 10^−5^ PE induced maximal contraction had no alternation, which means S1P incubation did not affect shortening velocity.

**Figure 8:**
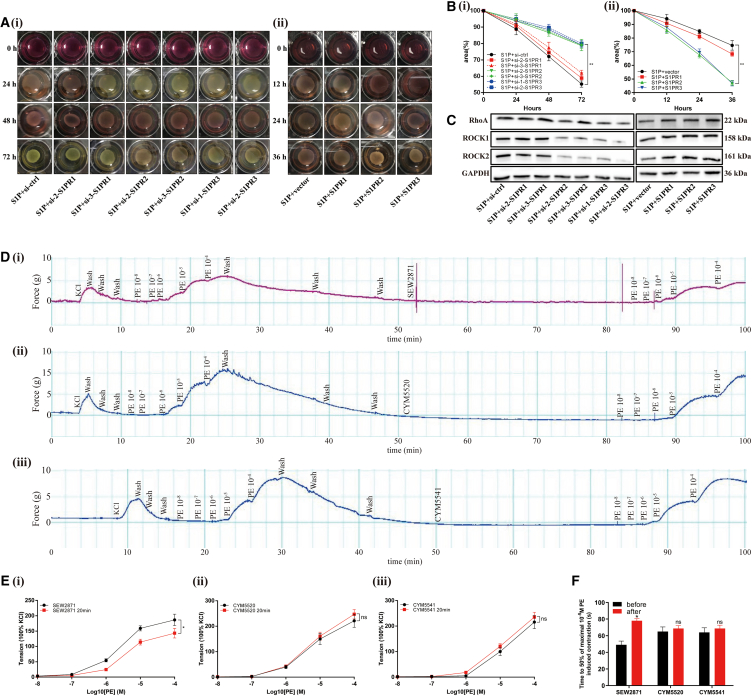
S1P/S1PR2/S1PR3 induced contraction of WPMY-1 cells and potentiated PE-mediated contraction of human prostate strips via RhoA/ROCK pathway (A) The effect of S1PRs knockdown and overexpression on contraction of WPMY-1 cells. (B) Statistical analysis of the percentages of the area of collagen gels. (C) Western blot assay of pathway-related proteins. (D) The effect of SEW2871, CYM5520 and CYM5541 on contraction of human prostate tissue strips. (E) Statistical analysis of tension (KCl = 100%). (F) Statistical analysis of time to 50% maximal 10^−5^ M PE induced contraction. Data are represented as M ± SD. ∗*p* < 0.05, ∗∗*p* < 0.01 and ns means no significant difference.

We also observed that the percent area of collagen gels with S1PR2-/S1PR3-knockdown was obviously amplified at the time point of 72 h (Figures 8A and 8B). In contrast, S1PR2 and S1PR3 overexpression led to strong decrease of the area of collagen gels at 12 h (Figures 8A and 8B). However, neither knockdown nor overexpression of S1PR1 had an effect on collagen gel size. In addition, we also found that the expression of RhoA, ROCK1, ROCK2 was downregulated with the knockdown of S1PR2 and S1PR3, while increased when S1PR2 and S1PR3 were overexpressed (Figure 8C). It was suggested that S1P/S1PR2/S1PR3 may modulate cell contraction via RhoA/ROCK pathway.

We further examined the involvement of S1PRs in the contraction of human normal prostate tissues via *in vitro* organ bath studies. We found that SEW2871 (S1PR1 agonist), CYM5520 (S1PR2 agonist), and CYM5541 (S1PR3 agonist) alone were not able to induce significant contraction at any of the various doses (data not shown). We then evaluated α1-adrenoreceptor-mediated contraction before and after incubation with these S1PR agonists. We demonstrated that PE (≥10^−6^ M) could induce concentration-dependent contraction of human prostate strips (Figure 8D). After washout, the second PE-induced contraction was significantly lowered at various dosages (data not shown). Interestingly, CYM5520 and CYM5541 incubation could prevent this tension loss, and even slightly increase tension generation, while SEW2871 did not show this effect (Figures 8D and 8E). In addition, human prostate strips exhibited a decreased shortening velocity in SEW2871 group after 20 min incubation, reflected by a longer time to 50% 10^−5^ M PE induced maximum contraction, while there was no significant change in CYM5520 group and CYM5541 group (Figure 8F).

### S1P/S1PR1/S1PR2/S1PR3 modulated inflammation via inhibiting the STAT3 pathway in prostate cells

**Figure 9:**
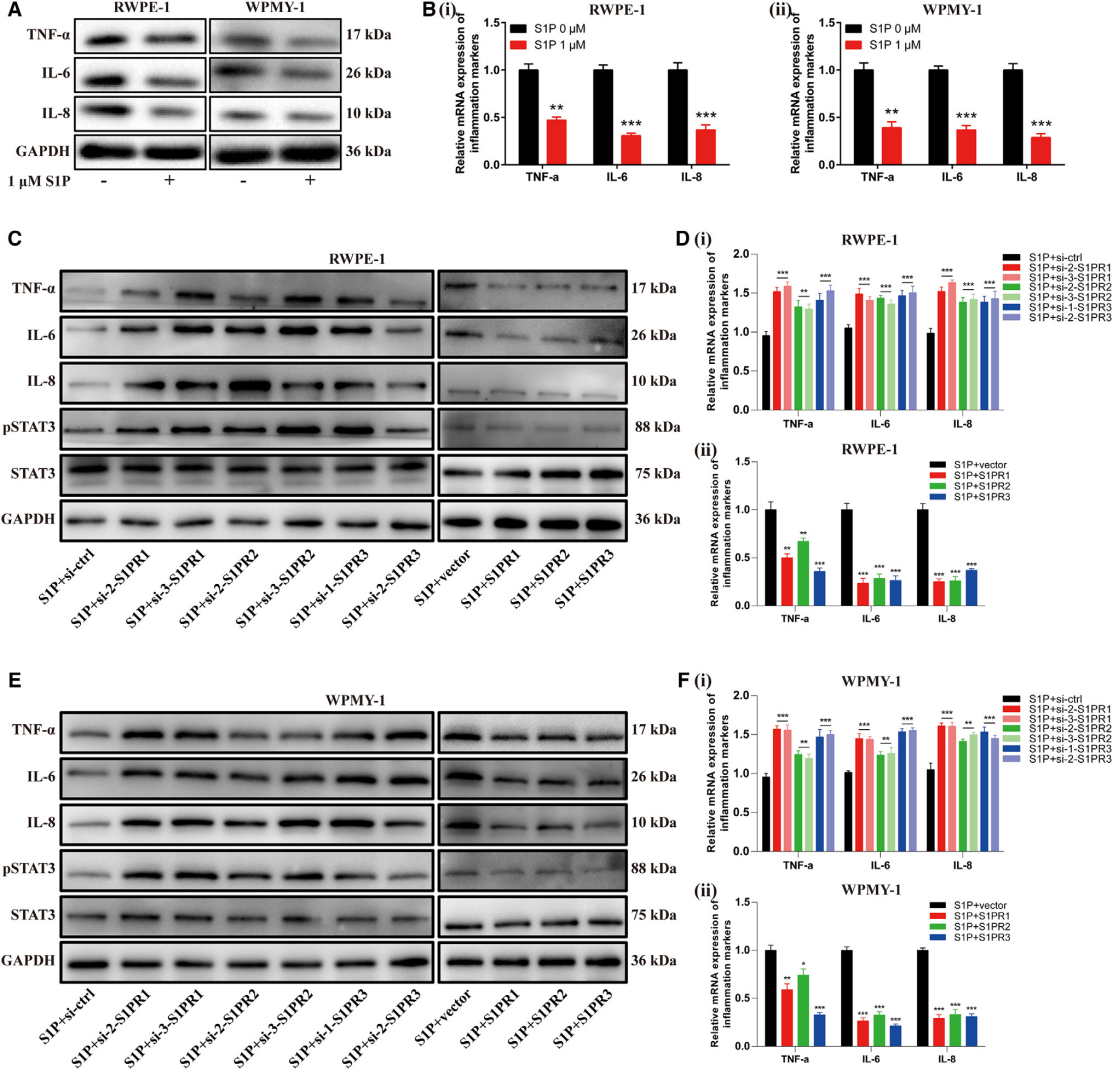
S1P/S1PR1/S1PR2/S1PR3 modulated inflammation via inhibiting STAT3 pathway in RWPE-1 and WPMY-1 cells (A and B) The effect of S1P on inflammation markers in RWPE-1 and WPMY-1 cells. (C–F) The protein and mRNA levels of inflammation markers after S1PR1/2/3 knockdown and overexpression in two cell lines. Data are represented as M ± SD. ∗∗*p* < 0.01 and ∗∗∗*p* < 0.001.

We examined the mRNA and protein levels of inflammatory markers (TNF-α, IL-6, IL-8) in prostate cells treated with S1P and found that S1P decreased their expression in the two cell lines (Figures 9A and 9B). We also investigated the effect of S1PRs on inflammation and found that both the mRNA and protein levels of inflammatory markers (TNF-α, IL-6, IL-8) increased by silencing S1PR1/2/3, while decreased with S1PR1/2/3 overexpression in two cell lines (Figures 9C–9F). Furthermore, knockdown of S1PR1/2/3 enhanced the phosphorylation of STAT3 and overexpression of S1PR1/2/3 decreased the phosphorylation of STAT3 (Figures 9A–C).

### S1P, SEW2871, and CYM5541 induced rat prostate enlargement *in vivo*

**Figure 10:**
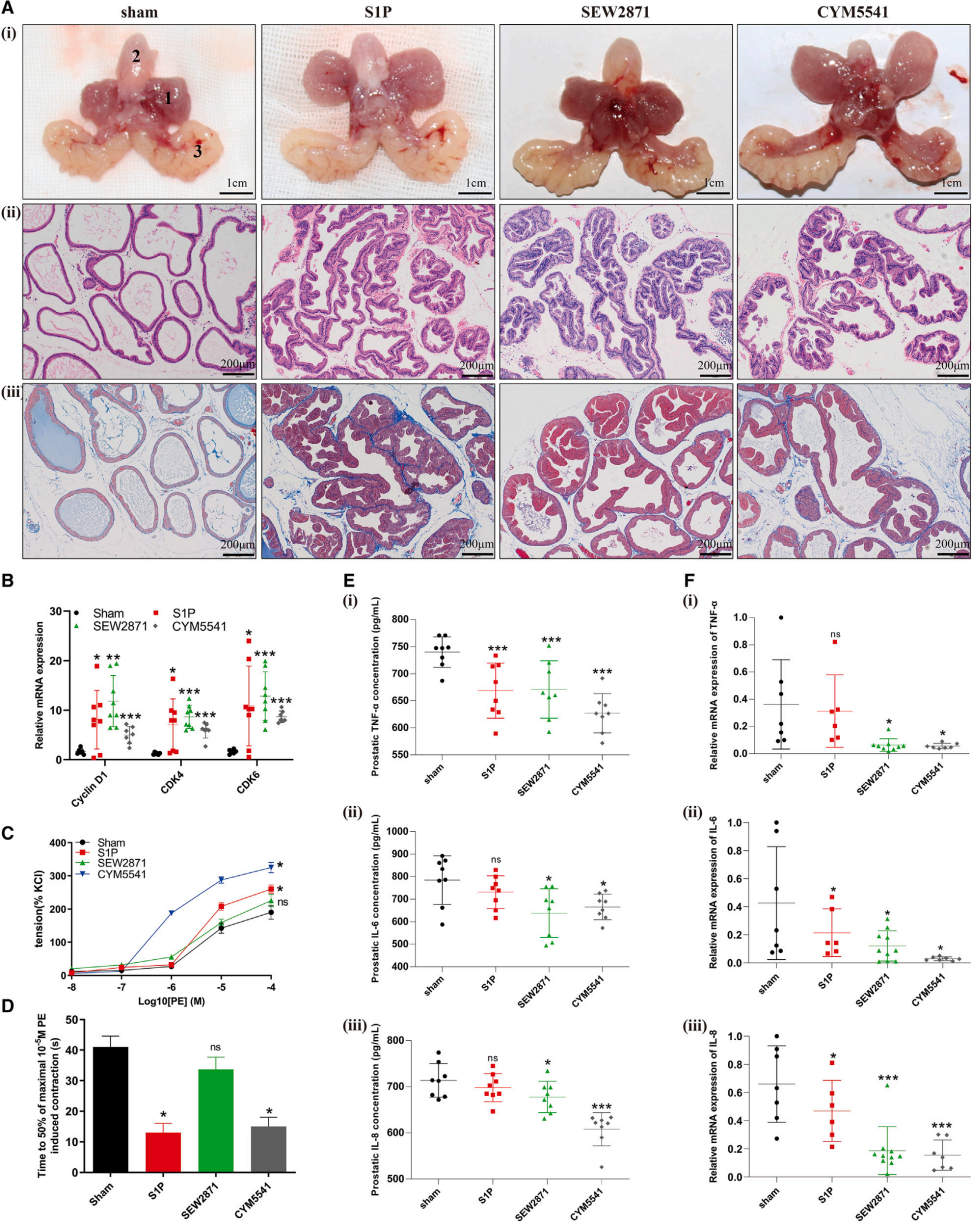
S1P, SEW2871, and CYM5541 induced BPH *in vivo* studies (A) The prostate tissues, H&E staining and Masson’s trichrome staining of sham, S1P-, SEW2871-, and CYM5541-treated rats. (1) ventral prostate, (2) bladder and (3) seminal vesicle. Representative graphs and micrographs are shown. The scale bars in (i) are 1 cm and in (ii-iii) are 200 μm. (B) Relative expression level of cell cycle markers (Cyclin D1, CDK4, CDK6) in rat prostate tissues. (C) Statistical analysis of tension in response to PE (compared to 100% KCl). (D) Statistical analysis of time to 50% maximal 10^−5^ M PE induced contraction. (E) Concentration of inflammation markers (TNF-α, IL-6, IL-8) in rat serum. (F) Relative expression level of inflammation markers (TNF-α, IL-6, IL-8) in rat prostate tissues. Data are represented as M ± SD. ∗*p* < 0.05, ∗∗*p* < 0.01, ∗∗∗*p* < 0.001 and ns means no significant difference.

We injected S1P, SEW2871, and CYM5541 into the bilateral ventral prostate of individual rats, respectively. As shown in Figure 10A and Table 6, the weight of the ventral prostate was obviously increased after 4 weeks treatment with S1P, SEW2871, and CYM5541, when compared with sham group. Additionally, prostate index (prostate wet weight [mg]/body weight [g]) was also upregulated in these groups (Table 6). Histologically, the structure of the rat prostate gland was clear, the glandular epithelia was a single layer in columnar arrangement and almost no epithelia was protruded into the lumen in the sham group, while the glandular epithelia were thickened significantly and the glands were protruded into the lumen in rats treated with S1P, SEW2871, and CYM5541 (Figure 10A). Additionally, rats treated with S1P, SEW2871, and CYM5541 showed an increased component of epithelia via Masson’s trichrome staining (Figure 10A). Consistent with the cellular level, S1P, SEW2871 and CYM5541 upregulated the expression of cell cycle markers (Cyclin D1, CDK4, CDK6) (Figure 10B). *In vitro* organ bath study revealed that the rat prostate, injected with S1P and CYM5541, exhibited stronger contractions and faster contraction velocities induced by PE (Figures 10C and 10D). Similar to the *in vitro* study, all inflammatory markers were downregulated both in rat serum and prostate tissues (Figure 10E and 10F).

**Table 6:** Variation of biometric and physiological parameters in rats

Group	Body weight(g)		Ventral prostate weight(mg)	Prostate index
	Initial	Final		
sham	337.63 ± 17.91	473.17 ± 37.80	598.74 ± 81.14	1.26 ± 0.25
S1P	341.75 ± 18.82	469.25 ± 39.67	798.32 ± 134.56^a^	1.81 ± 0.37^a^
SEW2871	335.13 ± 13.98	477.33 ± 34.64	771.11 ± 75.90^b^	1.70 ± 0.31^a^
CYM5541	343.25 ± 15.69	472.29 ± 26.61	725.75 ± 81.14^a^	1.62 ± 0.13^a^

a *p* < 0.05

b *p* < 0.01.

### W-146 and TY-52156 suppress testosterone-induced BPH *in vivo*

**Figure 11:**
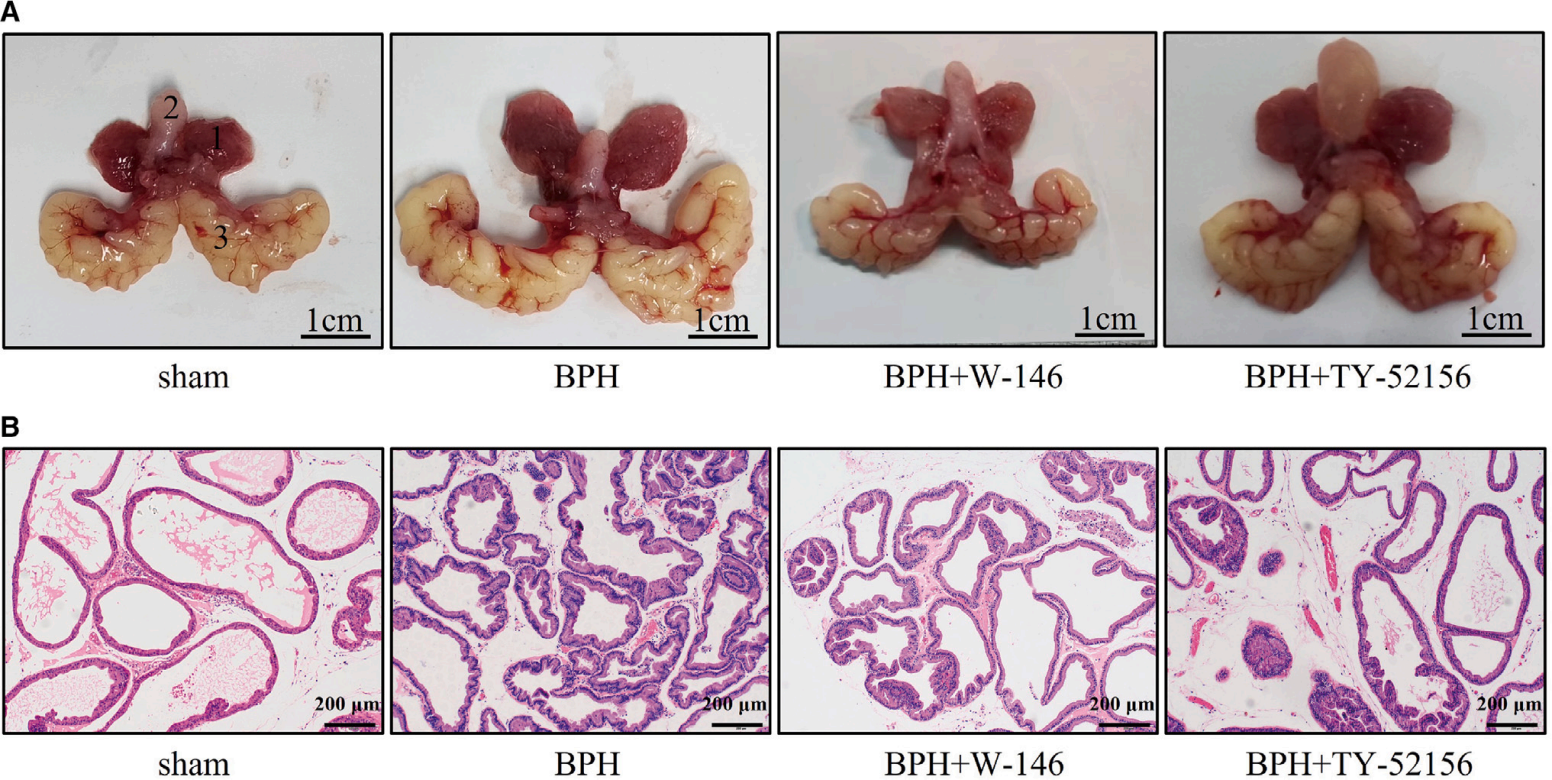
W-146 and TY-52156 suppress testosterone-induced BPH *in vivo* studies (A and B) The prostate tissues and H&E staining of sham, BPH, BPH+W-146, and BPH+TY-52156 rats. (1) ventral prostate, (2) bladder, and (3) seminal vesicle. Representative graphs and micrographs are shown. The scale bars in A are 1 cm and in B are 200 μm.

Finally, we confirmed the therapeutic effects of S1PR1/3 antagonists on the testosterone-induced rat BPH model. The weight of ventral prostate was obviously increased in the BPH rats (Figure 11A; Table 7). Interestingly, the BPH rats had a significant reduction in body weight during this experimental period (Table 7), which may be due to the physiological effect of testosterone. As shown in Figure 11A and Table 7, the weight of ventral prostate and prostate index were obviously decreased after 4 weeks treatment with W-146 and TY-52156, when compared with BPH group. Histologically, in BPH rats, the epithelium component was relatively increased, large glands lined with tall columnar epithelium, which were stratified, pseudostratified, or papillary fronds protruding into the lumen (Figure 11B). Nevertheless, W-146 and TY-52156 effectively prevented the progression of BPH induced by testosterone. The shrunk glands were lined with a single layer of columnar epithelium to low cuboidal cells, along with slight edema (Figure 11B).

**Table 7:** Variation of biometric and physiological parameters in rats

Group	Body weight(g)		Ventral prostate weight(mg)	Prostate index
	Initial	Final		
sham	313.56 ± 10.00	477.89 ± 20.70	596.56 ± 91.87	1.25 ± 0.17
BPH	312.63 ± 9.68	440.88 ± 17.63^b^	939.00 ± 74.94^c^	2.13 ± 0.17^c^
BPH+W-146	310.88 ± 15.83	439.88 ± 18.54	802.25 ± 73.74^d^	1.83 ± 0.23^a^
BPH+TY-52156	309.00 ± 16.38	446.63 ± 22.25	865.14 ± 52.35^d^	1.93 ± 0.16^a^

a *p* < 0.05 (BPH+W-146/BPH+TY-52156 vs. BPH).

b *p* < 0.01 (BPH vs. sham).

c *p* < 0.001 (BPH vs. sham).

d *p* < 0.01 (BPH+W-146/BPH+TY-52156 vs. BPH).

## Discussion

The current study demonstrated that both S1P and S1PR1/2/3 are abundantly expressed in the epithelium and stroma of the prostate, with almost no S1PR4/5 detected. We also showed that S1P could promote cell proliferation and cell contraction, while inhibiting inflammation in prostate cell lines. Moreover, our novel data revealed that S1P/S1PR1/S1PR3 promotes cell proliferation via AKT and ERK1/2 pathways, S1P/S1PR2/S1PR3 mediates contraction of prostate SM via RhoA/ROCK pathway, and that S1P/S1PR1/S1PR2/S1PR3 inhibits inflammatory response via the STAT3 pathway.

We found that the concentration of S1P in human normal prostate tissue was 26.86 ± 4.16 pmol/mg, which was within the range of that reported in prior studies.^31^ Previous studies indicated that S1PR1/2/3 are present in almost all tissues of mammalians, while S1PR4 is only expressed in lymphoid tissues and lung, and S1PR5 just in brain and skin.^5^^,^^12^ The various human and rat tissues we collected showed a similar distribution of S1PRs. As for the differences in S1PRs expression in different tissues between human and rat, it could be attributed to tissue specificity, physiological state, epigenetic modifications, RNA splicing and post-transcriptional modification, environmental factors, and others.^32^^,^^33^^,^^34^^,^^35^^,^^36^ Specifically, there are significant differences in physiological and biochemical characteristics between human and rat, which may lead to variations in the expression patterns and levels of S1PRs.^32^^,^^33^^,^^34^^,^^35^^,^^36^ With regard to prostate tissue, immunofluorescence microscopy revealed that S1PR1/2/3 were localized both in the epithelial and stromal compartments of rat and human prostate. In agreement with prostate tissue, S1PR1/2/3 were strongly expressed in two human prostate cell lines.

As we have known, S1P is involved in a wide array of biological processes, such as cell proliferation, survival, migration, inflammation, vasorelaxation, vasocontraction and others.^3^^,^^7^^,^^9^^,^^10^^,^^11^ However, its role in the prostate still remains unclear. This current study in the first to discover that S1P can promote cell proliferation and cell contraction, while inhibiting inflammation in human prostate cells. We further investigated, which individual S1PRs are specifically involved in those processes.

In our present study, we revealed that overexpression of S1PR1/3 enhanced, while knockdown of S1PR1/3 attenuated, S1P-induced cell proliferation *in vitro* with cell cycle associated-proteins (Cyclin D1, CDK4, and CDK6) altered accordingly. In line with our study, S1PR1/3 has been reported to enhance the proliferation of human amniotic cells and rat endothelial progenitor cells.^13^^,^^14^ In addition, several studies demonstrated that S1PR2 plays a role in proliferation of a variety of cell types.^18^^,^^19^^,^^20^^,^^21^ Actually, our current study found that S1PR2 could increase cell growth to a certain extent, but the change was not significant. Apart from cell proliferation, S1PRs were also observed to be involved in cell apoptosis. Downregulation of S1PRs induced apoptosis of multiple tumor cell types,^37^ while in non-neoplastic diseases, *Wang* et al. discovered that S1P attenuated apoptosis of endothelial progenitor cells induced by H_2_O_2_ via S1PR1/3^14^; *Liu* et al. showed that berberine (a chemical found in some plants) ameliorated ED in rats through the attenuation of apoptosis by inhibiting the SphK1/S1P/S1PR2 pathway.^38^ However, silence of S1PR1/3 showed no effect on the percentage of apoptotic cells in our two prostate cell lines.

S1PRs belong to the superfamily of G protein-coupled receptors (GPCRs). ERK and AKT are known to function in the downstream pathway of GPCRs, which are involved in cell proliferation and survival. Magnetic-field exposure was demonstrated to increase human amniotic cells proliferation mediated by the SphK/S1P/S1PR cascade via the ERK pathway.^13^ Also, S1P was found to amplify proliferation of endothelial progenitor cells via binding to S1PR1/3 to activate the PI3K/AKT pathway.^14^ Similarly, high-density lipoprotein enhanced proliferation of adipose-derived stem cells via S1PR1/AKT and ERK pathways.^16^ We also observed that S1PR1/3 overexpression promoted proliferation of prostate cells by increasing the phosphorylation of AKT and ERK. We further showed that alteration of proliferation could be reversed by U0126 and MK2206, which are specific antagonists of both signaling pathways, respectively. Therefore, S1P/S1PR1/S1PR3 may promote proliferation of prostate cells via the AKT and ERK pathways.

In addition to modulation of cell proliferation, our present study also demonstrates that S1P and S1PR1/2/3 can attenuate the inflammation response in prostate via the STAT3 pathway. FTY720 (fingolimod), as an effective modulator of S1PRs, has been demonstrated to exhibit protective effects on several autoimmune diseases.^39^ Its phosphorylated form, FTY720-P, shares homology with S1P and targets S1PR1/3/4/5. *Zhang* et al. generated an experimental autoimmune prostatitis (EAP) rat model via daily feeding of rats with FTY720, and found that FTY720 effectively suppressed development of EAP by dramatically reducing inflammatory infiltration of various immune cell populations into the rat prostate.^26^ In a variety of experimental autoimmune diseases, FTY720 induced the reduction of lymphocyte counts, inhibited the infiltration of lymphocytes and macrophages into target tissues and then prevented or attenuated the development of autoimmunity. Besides FTY720, other modulators of S1PRs such as AUY954 and SEW2871, also induce potent immunosuppressive and anti-inflammatory effects in different animal models.^40^^,^^41^ Drugs targeting S1PR1, such as fingolimod and siponimod, have been developed and are widely used in the clinical setting.^9^^,^^12^^,^^24^ With regard to S1PR2, a large proportion of studies revealed that the inhibition of S1PR2 can attenuate various inflammatory responses.^42^^,^^43^^,^^44^^,^^45^^,^^46^ On the other hand, some studies have indicated anti-inflammatory effects of S1PR2. *Michaud* et al. revealed an inhibitory role of S1PR2 in macrophage recruitment during inflammation^47^ while *Qu* et al. suggested that suppression of Th17 cell differentiation via S1PR2 by cinnamaldehyde can ameliorate ulcerative colitis.^48^ Additionally, S1PR3 was reported to promote inflammation.^49^^,^^50^^,^^51^^,^^52^^,^^53^ Moreover, the S1P/STAT3 pathway has been proven to mediate an inflammatory response.^54^^,^^55^

Consistent with earlier studies, the current study also suggests that S1P and S1PR2/3 plays pivotal roles in contraction of human stromal cell lines and human/rat prostate tissues. We observed that S1P and overexpression of S1PR2/3 led to a strong decrease while its knockdown induced an increase of the area of collagen gels with no vasoactive effect observed for S1PR1 interference. Moreover, S1P and agonists of S1PR1/3 could potentiate PE-triggered contraction of human prostate strips. Actually, S1P and all agonists of S1PRs alone showed no force generation for prostate tissues, but S1P and agonists of S1PR2/3 can prevent force loss during the second PE-induced contraction.

We also speculated that incubation of CYM5520 and CYM5541 may improve the fatigue of SM or increase the sensitivity of α1-adrenoreceptor. It is known that whether S1P mediates vasorelaxation or contraction depends on the species, the expression and localization of S1PR subtypes, and the physio-pathological state of the organism.^10^^,^^11^^,^^28^ In a previous study, we performed *in vitro* organ bath studies on rat corpus cavernosum SM (CCSM) and identified that agonizing S1PR1/3 induced CCSM contraction.^11^ *Kraft* et al. also corroborated the important role of FTY720 (S1PR1/3 agonist) in enhancing tone and contractility of rat gastric fundus SM.^56^ Additionally, the function of S1PR2 also has been investigated. *Pedowitz* et al. demonstrated that MYPT1 O-GlcNAc modification regulated S1P-mediated contraction of NIH3T3cells via activating S1PR2.^57^

Meanwhile, we have also provided clear evidence that antagonizing S1PR2 produced relaxation of CCSM.^11^ Similarly, *Liu* et al. found that JTE-013, an antagonist of S1PR2, partially improved erectile function by inhibiting SM contraction in rats.^58^ It has been demonstrated that S1P mediates SM contraction via RhoA/ROCK pathway.^59^^,^^60^^,^^61^ Contraction of SM is a complex process, intracellular Ca^2+^ combines with calmodulin (CAM) to form a Ca^2+^-CAM complex, then activates MLCK to phosphorylate MLC20. Ultimately, the Mg^2+^ ATPase in the head of *p*-MLC20 myosin regulatory light chains is activated to combine with actin and triggers contraction. The activated RhoA/ROCK pathway could increase the phosphorylation level of MLC directly. On the other hand, it also induced the phosphorylation of the MYPT1, which in turn inhibited the dephosphorylation of *p*-MLC.^59^^,^^62^^,^^63^ Therefore, we speculate that S1PR2/3 may promote contraction of prostate tissues via the RhoA/ROCK pathway.

Finally, we translated our *in vitro* studies into *in vivo* experiments. Considering that only S1P and S1PR1/3 agonists (SEW2871, CYM5541) were effective in promoting proliferation of prostate cells *in vitro*, we exclusively utilized S1P and S1PR1/3 agonists *in vivo*. In accordance with the cellular level, S1P and S1PR1/3 agonists increased the expression of cell cycle markers and decreased the levels of inflammation markers. Moreover, S1P and CYM5541 enhanced contraction of prostate tissues. Interestingly, S1P and S1PR1/3 agonists induced different degrees of hyperplasia and an increase of the prostate weight *in vivo*, while S1PR1/3 antagonist alleviated testosterone-induced BPH *in vivo*.

Collectively, this is the first study to show the expression and functional activities of S1P/S1PRs in the prostate. It indicates that S1P and S1PR1/2/3 are abundantly expressed in the prostate. It also suggests that S1P/S1PRs have a wide variety of functions, including modulation of cell proliferation, inflammation, and tissue tension. Except for the anti-inflammation role, all these processes would contribute to the development of BPH. Indeed, our *in vivo* study observed that intraprostatic injection of S1P and S1PR1/3 agonists induced experimental BPH, while S1PR1/3 antagonist alleviated testosterone-induced BPH. A number of the S1PR agonists like fingolimod (FTY720), siponimod, ponesimod and ozanimod are S1PR1 modulators, have been approved by FDA for various forms of multiple sclerosis. Therefore, S1P/S1PRs may be rediscovered as a promising therapeutic target for the treatment of BPH.

### Limitations of the study

Firstly, this study demonstrated that S1P/S1PRs play a critical role in the development of BPH by regulating cell proliferation, contraction, and inflammatory responses through the AKT, ERK1/2, RhoA/ROCK, and STAT3 pathways. However, these mechanisms may not fully explain the role of S1P/S1PRs, as other signaling pathways could also be involved. Secondly, apart from the inhibitors of AKT and ERK1/2, the study did not evaluate the effects of inhibitors targeting other pathways, resulting in relatively limited evidence. Furthermore, the direct targets or additional potential mechanisms of S1P/S1PRs in BPH remain to be explored. Given the promising therapeutic effects of S1P/S1PRs on BPH observed in rats, further research is warranted.

## Resource availability

### Lead contact

Further information and requests for reagents and resources should be directed to and will be fulfilled by the lead contact, Xinhua Zhang (zhangxinhuad@163.com).

### Materials availability

The study did not generate new unique reagents.

### Data and code availability

• All data reported in this paper will be shared by the lead contact upon reasonable request.
• This paper does not report original code.
• Any additional information required to reanalyze the data reported in this paper is available from the lead contact upon request.

## Acknowledgments

This study was supported in part by 10.13039/501100001809National Natural Science Foundation of China N.82070780. We acknowledge the help of the staff at Zhongnan Hospital of Wuhan University in completing the study.

## Author contributions

D.L., J.L., Y.L., and L.D. contributed equally to this work. X.Z., D.L., J.L., Y.L., and L.D. designed and finished the experiments. D.L., J.L., Y.L., and L.D. wrote the first draft. D.L., J.L., Y.L., L.D., Q.C., L.Y., Y.Z., P.C., Y.G., G.Z., and W.H. analyzed the results and prepared all figures and tables. M.E.D., W.H., and X.Z. critically revised drafts of the manuscript. X.Z. provided important intellectual input and approved the final version for publication. All authors reviewed the manuscript.

## Declaration of interests

The authors declare no competing interests.

## STAR★Methods

### Key resources table

**Table:** 

REAGENT or RESOURCE	SOURCE	IDENTIFIER
**Antibodies**		

See Tables 3 and 4	This paper	N/A

**Biological samples**		

Human bladder, kidney, liver, lung, lymph node, prostate, sperm duct, spleen, testis, ureter, vessel tissues and serum	Zhongnan Hospital, Wuhan University	N/A
Rat bladder, brain, heart, kidney, liver, lung, penis, prostate, spleen, testis and vessel tissues	Normal male Sprague-Dawley rats	N/A

**Chemicals, peptides, and recombinant proteins**		

S1P	Sigma	Cat. 73914
U0126	MCE	Cat. HY-12031A
MK2206	MCE	Cat. HY-108232
SEW2871	MCE	Cat. HY-W008947
CYM5520	Topscience	Cat. T22703
CYM5541	MCE	Cat. HY-101419
W-146	MCE	Cat. HY-101395
TY-52156	MCE	Cat. HY-19736

**Critical commercial assays**		

Hipure Total RNA Mini Kit	Magen	Cat. R4111-03
ABScript II RT Master Mix for qPCR	Abclonal	Cat. RK20402
2× Universal SYBR Green Fast qPCR Mix	Abclonal	Cat. RK21203
Cell cycle staining kit	Multi Sciences	Cat. CCS012
Annexin V-FITC/PI apoptosis kit	Multi Sciences	Cat. AP101-100-kit
Cell counting kit-8	Meilun Biotechnology	Cat. MA0218-L
Cell Contraction Assay Kit	Cell Biolabs	Cat. CBA-201
Human S1P ELISA kit	Meimian	Cat. MM-91866O1
rat IL-6 ELISA kit	Meimian	Cat. MM-0190R2
rat IL-8 ELISA kit	Meimian	Cat. 0175R2
rat TNF-αELISA kit	Meimian	Cat. MM-0180R2

**Experimental models: Cell lines**		

RWPE-1	ATCC	Cat. CRL11609
WPMY-1	Stem Cell Bank	Cat. GNHu36

**Experimental models: Organisms/strains**		

Sprague-Dawley rat	This paper	N/A

**Oligonucleotides**		

si-RNA, see Table 5	Genepharma	N/A
Primer for RT-qPCR, see Tables 1 and 2	This paper	N/A

**Recombinant DNA**		

S1PR1, S1PR2, S1PR3	Fenghui	Cat. JY3307, Cat. JY4554, Cat. JY4555

**Software and algorithms**		

Adobe Illustrator	Adobe	https://www.adobe.com/
ImageJ	NIH	https://imagej.nih.gov/ij/
Prism	Graphpad	https://www.graphpad.com

### Experimental model and study participant details

#### Collection of human samples

Human bladder, kidney, liver, lung, lymph node, prostate, sperm duct, spleen, testis, ureter, vessel tissues and serum were collected from young male brain-dead organ donors (*n* = 10, 28.2 ± 4.4 years) undergoing organ donation at the Organ Transplant Center of Zhongnan Hospital. All tissues were normal without tumor or other disease based upon pathological examination. All samples except prostate and serum were divided into two parts: some tissues were frozen in liquid nitrogen for RT-qPCR and Western-blot, or stored in 10% neutral buffered formalin for histological examination. As for the prostate, about 2 × 2 × 10 mm prostatic strips were prepared for organ bath studies and immediately placed in Krebs buffer. The remaining tissues were frozen in liquid nitrogen for RT-qPCR and Western-blot, or stored in 10% neutral buffered formalin for histological examination. Serums were frozen in liquid nitrogen for the detection of S1P via ELISA. Since the prostate is a male-specific organ, there is no influence of gender on the results of the study. All human studies were conducted in accordance with the principles of the Declaration of Helsinki and all human samples were obtained after approval of the Ethics Committee of Zhongnan Hospital, Wuhan University (Approval code: 2021038).

#### Animals

Specific-pathogen-free (SPF) grade male Sprague–Dawley rats (8 weeks old) weighing 200–250 g were purchased from Beijing Vital River Laboratory Animal Technology Co., Ltd. Since the prostate is a male-specific organ, there is no influence of gender on the results of the study. Animal experiments were conducted at the Animal Center of Zhongnan Hospital, Wuhan University and all animal protocols were approved by the Medical Ethics Committee for Experimental Animals of Zhongnan Hospital, Wuhan University (Approval code: AF248).

#### Collection of rat tissues

Rat bladder, brain, heart, kidney, liver, lung, penis, prostate, spleen, testis and vessel tissues were harvested from normal male Sprague-Dawley rats (*n* = 10). Some tissues were frozen in liquid nitrogen for RT-qPCR and Western-blot, or stored in 10% neutral buffered formalin for histological examination.

#### Intraprostatic injection of S1P, SEW2871 and CYM5541 in normal rats

Thirty-two male Sprague-Dawley rats were used for the *in vivo* treatment studies. All rats were divided into four groups: sham, S1P, SEW2871 and CYM5541 treated, after a week-long period of acclimatization. After anesthetizing using sodium pentobarbital and disinfecting with iodine, a surgical midline incision of the lower abdomen above the penis was made to expose the ventral prostate. Stock solutions of S1P (Sigma-Aldrich, Shanghai, China, Cat. 73914), SEW2871 (MCE, Monmouth Junction, NJ, USA, Cat. HY-W008947) and CYM5541 (MCE, Cat. HY-101419) were prepared in dimethyl sulfoxide (DMSO) and then 0.5 nmol of S1P, SEW2871 and CYM5541 in a final volume of 50 μL sterile normal saline were injected into both right and left ventral lobes of the prostate. For the sham group, 50 μL of sterile normal saline with nearly commensurable DMSO was injected. A 2% lidocaine solution was applied to the wound after the injection and then the wound was closed. Rats were euthanized 4 weeks post-surgery and then blood was collected from the heart for ELISA assay. Moreover, ventral prostate, bladder, and seminal vesicle were harvested and weighed. About 2 × 2 × 10 mm prostatic strips were prepared for organ bath studies and immediately placed in Krebs buffer. The remaining tissues were frozen in liquid nitrogen for RT-qPCR and Western-blot, or stored in 10% neutral buffered formalin for histological examination.

#### Intraprostatic injection of W-146 and TY-52156 in rat BPH model

Thirty-two male Sprague-Dawley rats weighing 200–250 g were used for the *in vivo* treatment studies. The method for constructing the BPH rat model using testosterone propionate is described in our previous study.^64^ Specifically, 2 mg of testosterone propionate was injected subcutaneously daily for 4 weeks to build rat BPH model. All rats were divided into four groups: sham, BPH, BPH+W-146, BPH+TY-52156. After anesthetizing using sodium pentobarbital and disinfecting with iodine, a surgical midline incision of the lower abdomen above the penis was made to expose the ventral prostate. Stock solutions of W-146 (MCE, Monmouth Junction, NJ, USA, Cat. HY-101395) and TY-52156 (MCE, Monmouth Junction, NJ, USA, Cat. HY-19736) were prepared in dimethyl sulfoxide (DMSO) and then 0.5 nmol of W-146 and TY-52156 in a final volume of 50 μL sterile normal saline were injected into both right and left ventral lobes of the prostate. For sham and BPH group, 50 μL of sterile normal saline with nearly commensurable DMSO was injected. A 2% lidocaine solution was applied to the wound after the injection and then the wound was closed. Rats were euthanized 4 weeks post-surgery. Ventral prostate, bladder and seminal vesicle were harvested and stored in 10% neutral buffered formalin for histological examination.

#### Cell culture

Human normal prostate epithelial cell line RWPE-1 (Cat. CRL11609) was obtained from the American Type Culture Collection in Manassas, Virginia, USA and SV40 large-T antigen-immortalized stromal cell line WPMY-1 (Cat. GNHu36) was purchased from the Stem Cell Bank, Chinese Academy of Sciences in Shanghai, China. RWPE-1 cells were maintained in KSF medium supplemented with EGF and BPE (Invitrogen, Waltham, MA, USA). WPMY-1 cells were cultured in DMEM medium containing 1% penicillin G sodium/streptomycin sulfate and 5% FBS (Gibco, Waltham, Massachusetts, USA). All cells were cultured in a humidified atmosphere consisting of 95% air and 5% CO_2_ at 37°C. All cell lines were identified by the China Center for Type Culture Collection and tested for mycoplasma contamination.

### Method details

#### Addition of exogenous S1P

After treated with S1P at 0, 0.1, 0.5, 1 μM, CCK-8 assay was used to detect cell proliferation and identify the optimal S1P concentration (1 μM). This concentration was used for all subsequent experiments.

#### Knockdown of S1PR1/2/3

S1PR1-, S1PR2-and S1PR3-target specific small interfering RNAs (siRNAs) were synthesized by Genepharma Co., Ltd. in Suzhou, China. The sequences of siRNAs are shown in Table 5 siRNA transfection was performed using the siRNA transfection reagent (Genepharma, Suzhou), according to the manufacturer’s instructions. The original medium was replaced by the complete medium and 1 μM S1P was added after 6 h. After transfection for 48 h, the transfected cells were harvested for the following experiments.

#### Overexpression of S1PR1/2/3

The S1PR1/2/3 plasmids (Cat. JY3307, Cat. JY4554, Cat. JY4555) were purchased from Fenghui Biotechnology Co., Ltd. in Hunan, China. Transfection was performed according to the manufacturer’s instructions. The original medium was replaced by the complete medium and 1 μM S1P was added after 6 h. After transfection for 48 h, the transfected cells were harvested for the following experiments.

#### Addition of U0126 and MK2206

ERK1/2 pathway inhibitor U0126 (Cat. HY-12031A) and AKT pathway inhibitor MK2206 (Cat. HY-108232) were purchased from MCE. After transfection with S1PRs plasmids for 24 h, U0126 (10 μM)^65^ and MK2206 (10 μM)^66^ were added to cotreat transfected cells for 24 h, respectively.

#### Total RNA extraction, mRNA reverse transcription and RT-qPCR analysis

Total RNA was extracted according to the instructions of Hipure Total RNA Mini Kit (Magen, Guangdong, China, Cat. R4111-03), RNA reverse transcription was performed using ABScript II RT Master Mix for qPCR (Abclonal, Wuhan, China, Cat. RK20402) and RT-qPCR was conducted in accordance with the instruction manual of 2× Universal SYBR Green Fast qPCR Mix (Abclonal, Cat. RK21203). All samples were run in triplicate and independently repeated three times. Gene expression levels were normalized to GAPDH mRNA and calculated by 2^−ΔΔCT^ method. All primer sequences are listed in Tables 1 and 2.

#### Western-blot analysis

Total proteins extracted from prostate cells and tissues were separated by 10% SDS-PAGE and transferred to PVDF membranes. After blocking with 5% non-fat milk, the membranes were incubated sequentially with primary and secondary antibodies (listed in Tables 3 and 4). Finally, the bands on membranes were visualized by ECL reagents. The relative expression levels of target proteins were normalized to GAPDH. All samples were independently repeated three times and means were calculated.

#### Flow cytometry analysis

A cell cycle staining kit (Cat. CCS012) and Annexin V-FITC/PI apoptosis kit (Cat. AP101-100-kit) were obtained from Multi Sciences Co., Ltd. in Hangzhou, China). For cell cycle analysis, the harvested cells were incubated with DNA staining solution and permeabilization solution to analyze the DNA content distribution. For cell apoptosis, the treated cells were collected and then incubated with Annexin V-FITC/PI apoptosis kit.

#### Cell proliferation assay

Cell counting kit-8 (Cat. MA0218-L), obtained from Meilun Biotechnology Co., Ltd. in Dalian, China, was used to detect cell proliferation. Briefly, RWPE-1 and WPMY-1 cells (3000 cells/well) were plated in 96-well microtiter plates and CCK-8 reagents were added to each well at different times. After incubation for 1 h, absorbance was measured at a wavelength of 450 nm.

#### Immunofluorescent staining for cells

Coverslips containing RWPE-1 or WPMY-1 cells were fixed with 4% PFA (paraformaldehyde) and permeabilized with 0.1% Triton X-100. After blocking with normal goat serum, primary antibodies (listed in Table 3) and Cy3-labeled or FITC-labeled secondary antibodies (listed in Table 4) were incubated sequentially. Finally, DAPI was added to stain the nuclei. Visualization was performed on a Laser Scanning Confocal Microscope (Olympus, Japan).

#### Immunofluorescent staining of tissues

Frozen tissues were sectioned in 10 μm thick slices and thawed, mounted onto glass slides, air-dried, and fixed in ice-cold acetone. After incubation in a mixture of PBS supplemented with Triton X-100 and bovine serum albumin, primary antibodies (listed in Table 3) and Cy3-labeled or FITC-labeled secondary antibodies (listed in Table 4) were incubated sequentially. Finally, DAPI was added to stain the nuclei. Visualization was performed on a Laser Scanning Confocal Microscope (Olympus, Japan).

#### Hematoxylin and eosin (H&E) staining

Paraffin sections were deparaffinized, rehydrated, then stained with 10% Hematoxylin and 1% Eosin containing 0.2% glacial acetic acid, sequentially. Finally, the sections were imaged by an Olympus-DP72 light microscope (Olympus, Japan).

#### Masson’s trichrome staining

Paraffin sections were stained using Masson’s trichrome staining. Prostatic SM cells, collagen fibers, and epithelial cells were stained red, blue, and orange, respectively. The percent area of SM, collagen fibers, and glandular epithelium were quantitated with ImageJ software (NIH, USA).

#### Collagen-based cell contraction assay

Cell Contraction Assay Kit (Cat. CBA-201) was purchased from Cell Biolabs, Inc. (San Diego, CA, USA) and was used to test the contractility of WPMY-1 cells according to the manufacturer’s protocol. Firstly, the pre-treated WPMY-1 cells were harvested, resuspend and adjusted to a cell density of 2–5 × 10^6^ cells/mL. The collagen lattice was prepared by mixing 2 parts of cell suspension and 8 parts of cold Collagen Gel Working Solution. Secondly, 0.5 mL of the cell-collagen mixture was added to each well in a 24-well plate and incubated for 1 h at 37°C to polymerize the collagen matrix. Next, 1.0 mL of DMEM medium was added to each collagen gel lattice. Finally, the cell cultures were incubated for two days to develop stress. To initiate contraction, collagen gels were gently released from the sides of the culture dishes with a sterile spatula. The size of the collagen gel was measured at various times with a ruler and the percentage of the area of collagen gel to the original area was calculated.

#### *In vitro* organ bath studies

Human and rat prostate strips were mounted longitudinally in 10 mL ZW-SX digital thermostats smooth groove (Wuxi Woshin Instruments Manufacturing Co., LTD, Jiangsu, China) which was filled with 10 mL Krebs buffer at 37°C and constantly bubbled with 95% O_2_ and 5% CO_2_. The Krebs buffer had the following mM composition: NaCl 110, KCl 4.8, CaCl_2_ 2.5, MgSO_4_ 1.2, KH_2_PO_4_ 1.2, NaHCO_3_ 25 and dextrose 11. One pin of the strips was connected to a force transducer that was attached to a PowerLab 4/30 data acquisition system (Shanghai, China) to record the tension of prostate tissues. Strips were equilibrated at least 1 h and Krebs buffer was changed every 15 min. The initial resting tension of strips were continuously adjusted to 600 mg for rat prostate and 1500 mg for human prostate. After equilibration, strips were precontracted with 60 mM KCl and then washed followed by contracting with increasing concentrations (10^−8^-10^−4^ M) of phenylephrine (PE). For human prostate, strips were washed and incubated with S1P, SEW2871, CYM5520 (S1PR2 agonist, Topscience, Cat. T22703) and CYM5541 for 20 min, respectively, after stimulating by 10^−4^ M PE. Thereafter, strips were contracted with increasing concentrations (10^−8^-10^−4^ M) of PE again. Force produced by the aforementioned stimuli was normalized to tension (% KCl).

#### Enzyme-linked immunosorbent assay (ELISA)

Human S1P (Cat. MM-91866O1), rat IL-6 (Cat. MM-0190R2), rat IL-8 (Cat. 0175R2) and rat TNF-α (Cat. MM-0180R2) ELISA kits were purchased from Jiangsu Meimian Industrial Co., Ltd, China. According to the manufacturer’s instructions, S1P concentration in human prostate tissue and concentration of IL-6, IL-8, TNF-α in rat serum were determined by ELISA.

### Quantification and statistical analysis

All data in the experiment were biologically replicated at least three times and presented as mean ± standard deviations. Student’s two-tailed t-test (for two groups) and one-way ANOVA (for multiple groups) were used to compare the means. *p* < 0.05 was considered statistically significant.
