# Supplementary material for: Broad and diverse roles of sphingosine-1-phosphate/sphingosine-1-phosphate receptors in the prostate
Source: iScience. 2024 Oct 30;27(12):111290. doi: 10.1016/j.isci.2024.111290 (PMC11607605; doi:10.1016/j.isci.2024.111290)
Supplement: Document S1. Figures S1–S7 and Data S1–S7 [file mmc1.pdf]

iScience, Volume *27*

Supplemental information

Broad and diverse roles of sphingosine-1-phosphate/sphingosine-1-phosphate receptors in the prostate

Daoquan Liu, Jianmin Liu, Yan Li, Lu Du, Qingqiong Cao, Liang Yang, Yongying Zhou, Ping Chen, Yuming Guo, Guang Zeng, Michael E. DiSanto, Weidong Hu, and Xinhua Zhang

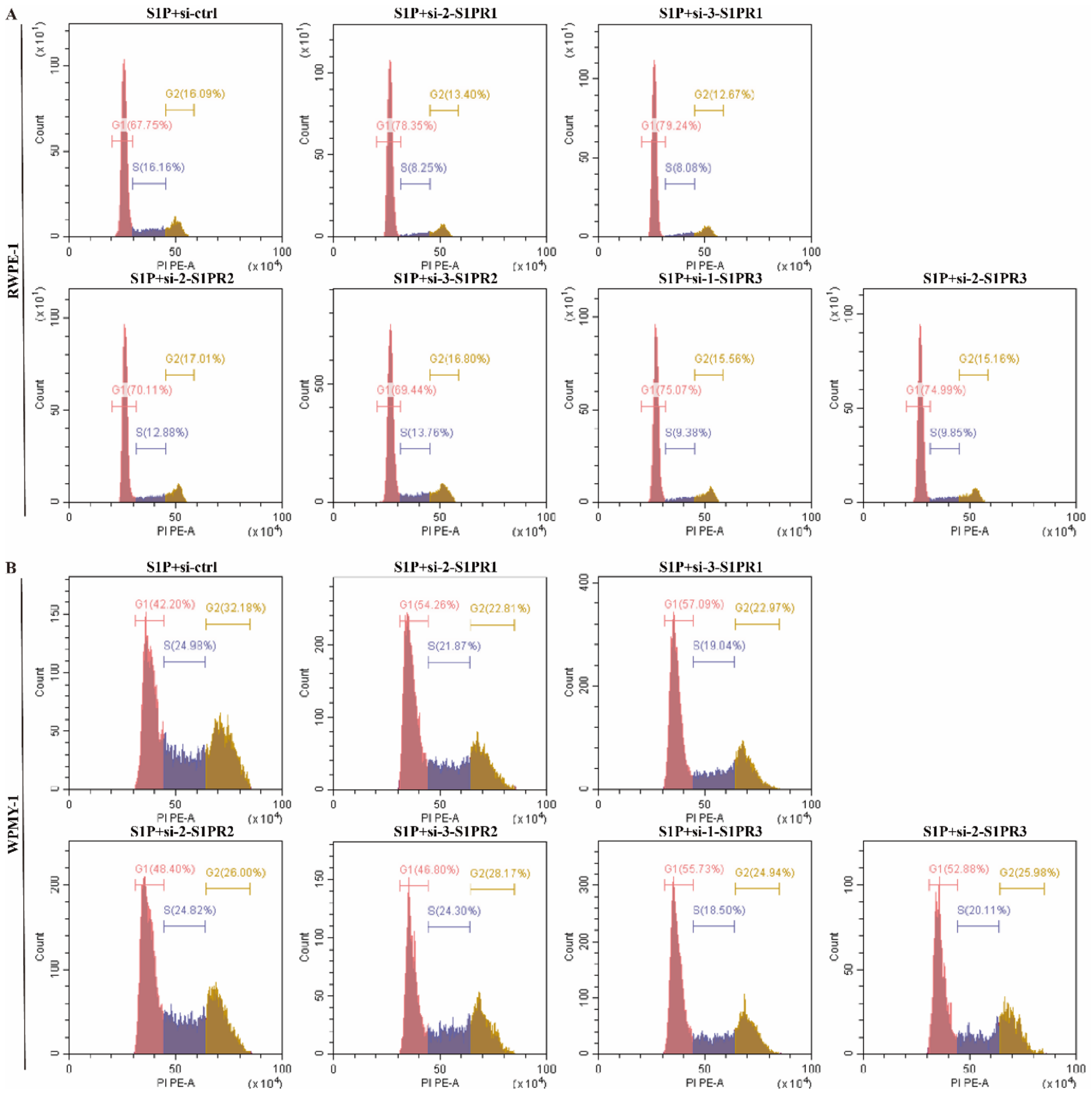

Fig S1. The effect of S1PR1/2/3 knockdown on cell cycle of RWPE-1 and WPMY-1 cells.

(A) The effect of S1PR1/2/3 knockdown on cell cycle of RWPE-1 cells. **(B)** The effect of S1PR1/2/3 knockdown on cell cycle of WPMY-1 cells.

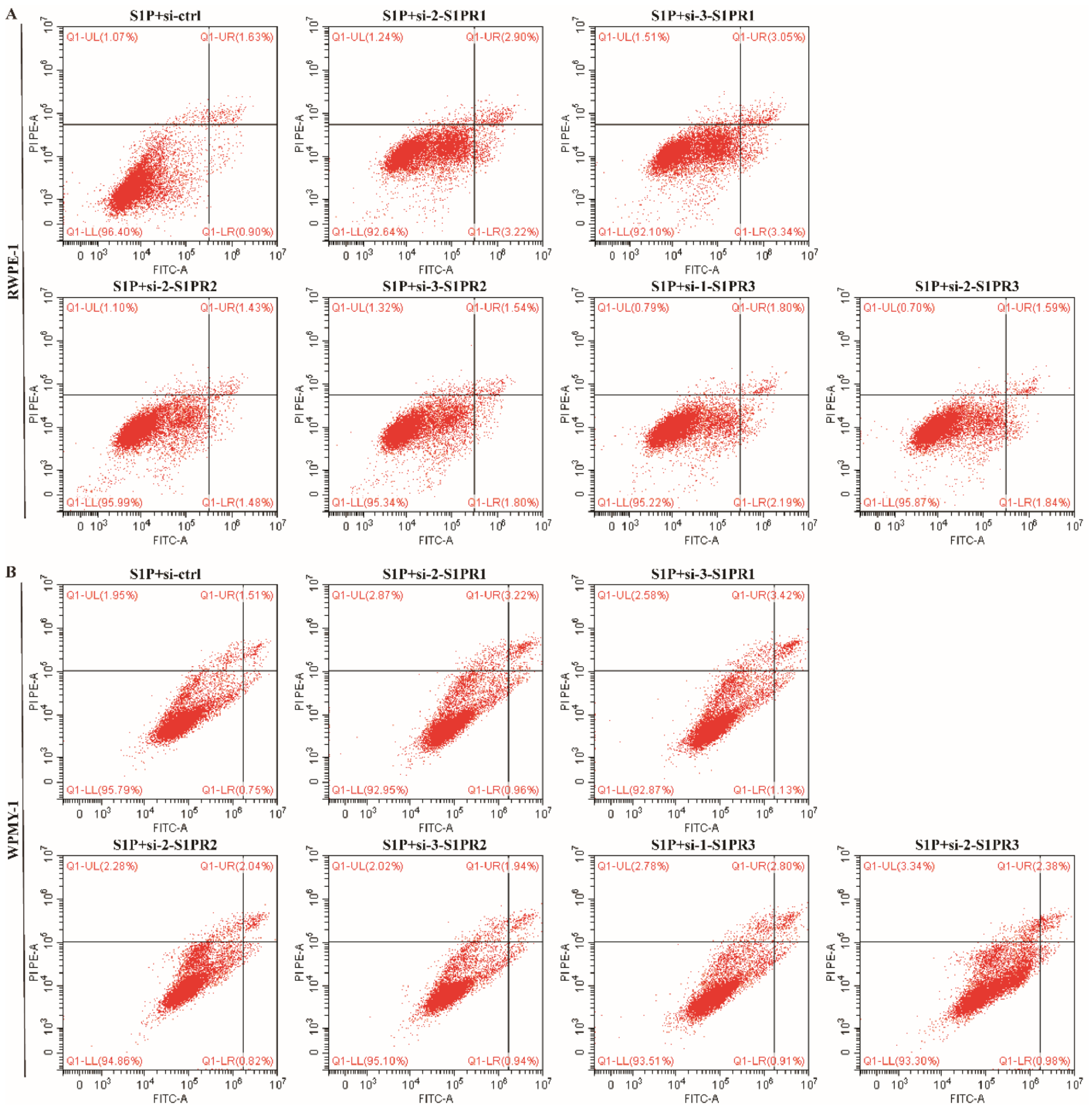

Fig S2. The effect of S1PR1/2/3 knockdown on apoptosis of RWPE-1 and WPMY-1 cells.

(A) The effect of S1PR1/2/3 knockdown on apoptosis of RWPE-1 cells. **(B)** The effect of S1PR1/2/3 knockdown on apoptosis of WPMY-1 cells.

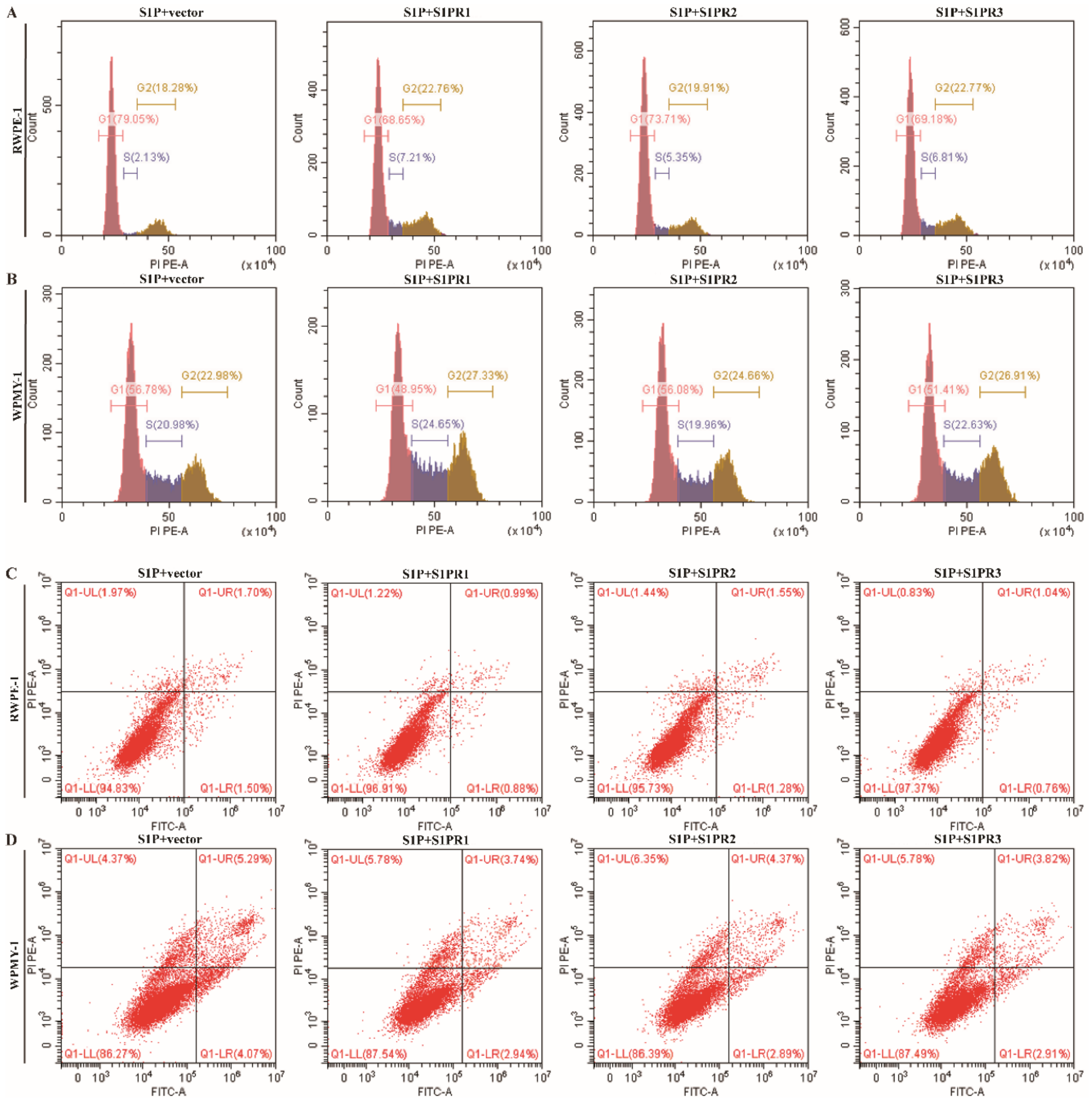

Fig S3. The effect of S1PR1/2/3 overexpression on cell cycle and cell apoptosis of RWPE-1 and WPMY-1 cells.

(A) The effect of S1PR1/2/3 overexpression on cell cycle of RWPE-1 and WPMY-1 cells. **(B)** The effect of S1PR1/2/3 overexpression on cell apoptosis of RWPE-1 and WPMY-1 cells.

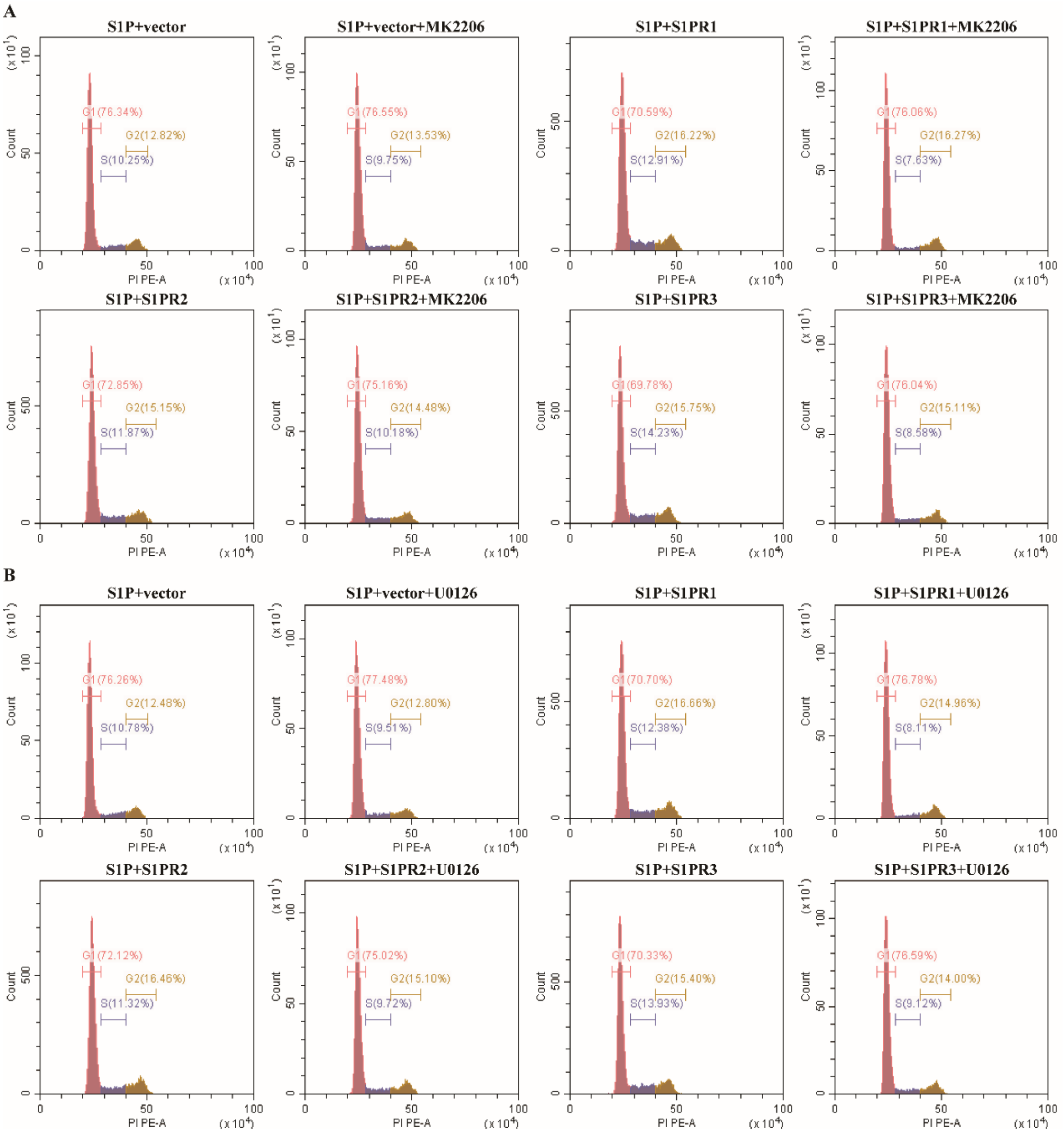

Fig S4. The effect of MK2206 and U0126 on cell cycle of RWPE-1 cells.

(A) The effect of MK2206 on cell cycle of RWPE-1 cells. **(B)** The effect of U0126 on cell cycle of RWPE-1 cells.

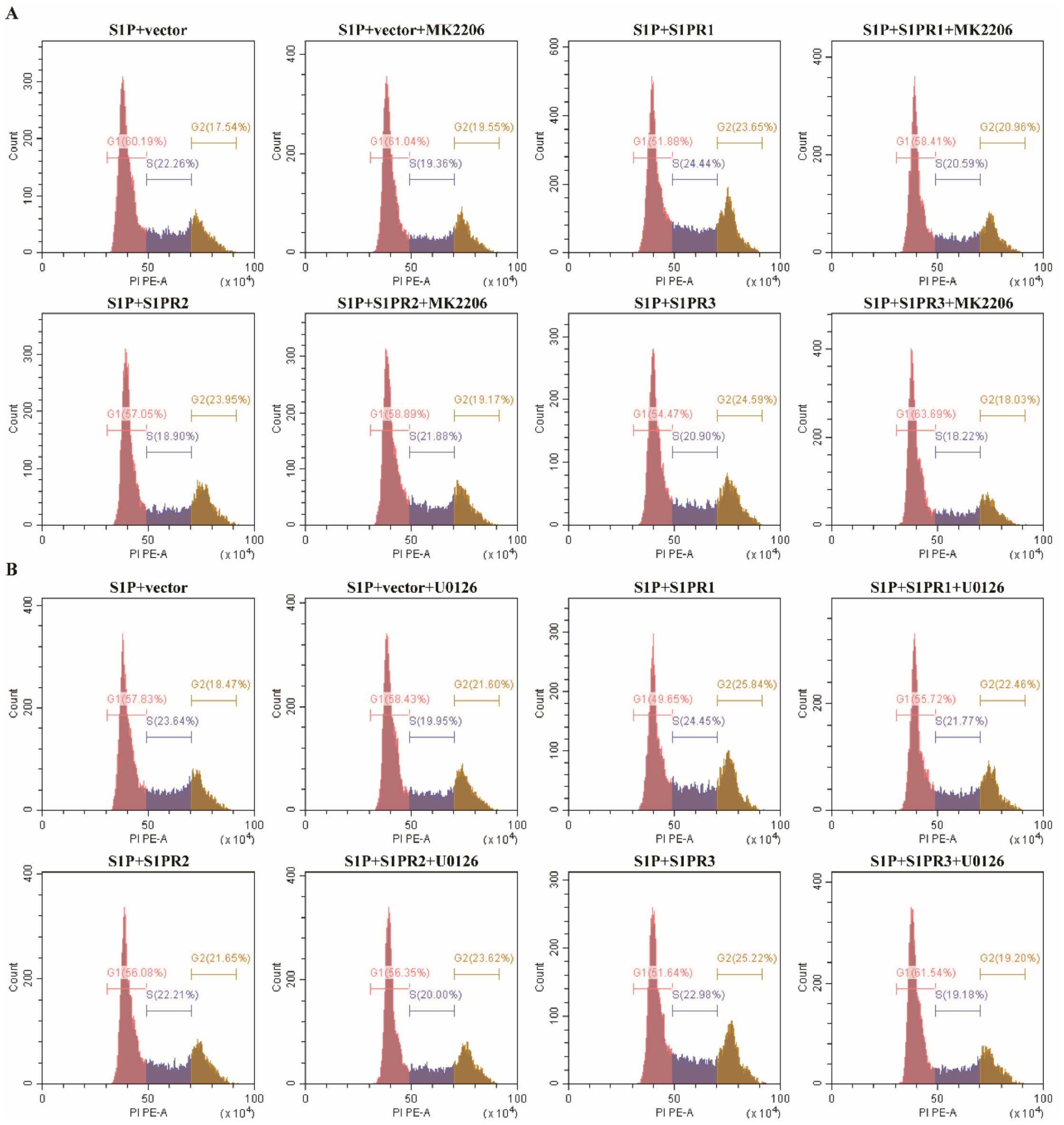

Fig S5. The effect of MK2206 and U0126 on cell cycle of WPMY-1 cells.

(A) The effect of MK2206 on cell cycle of WPMY-1 cells. **(B)** The effect of U0126 on cell cycle of WPMY-1 cells.

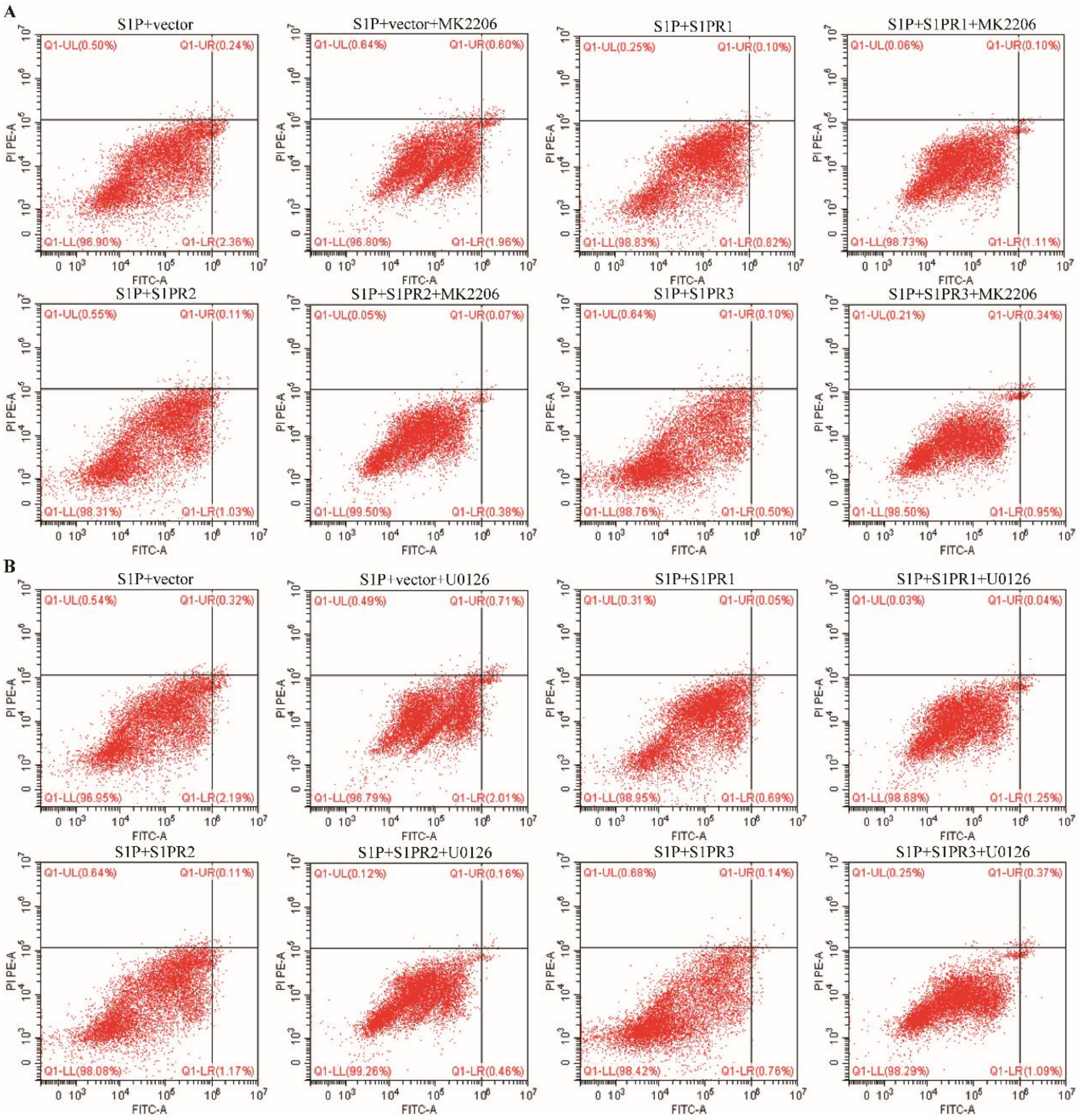

Fig S6. The effect of MK2206 and U0126 on apoptosis of RWPE-1 cells.

(A) The effect of MK2206 on apoptosis of RWPE-1 cells. **(B)** The effect of U0126 on apoptosis of RWPE-1 cells.

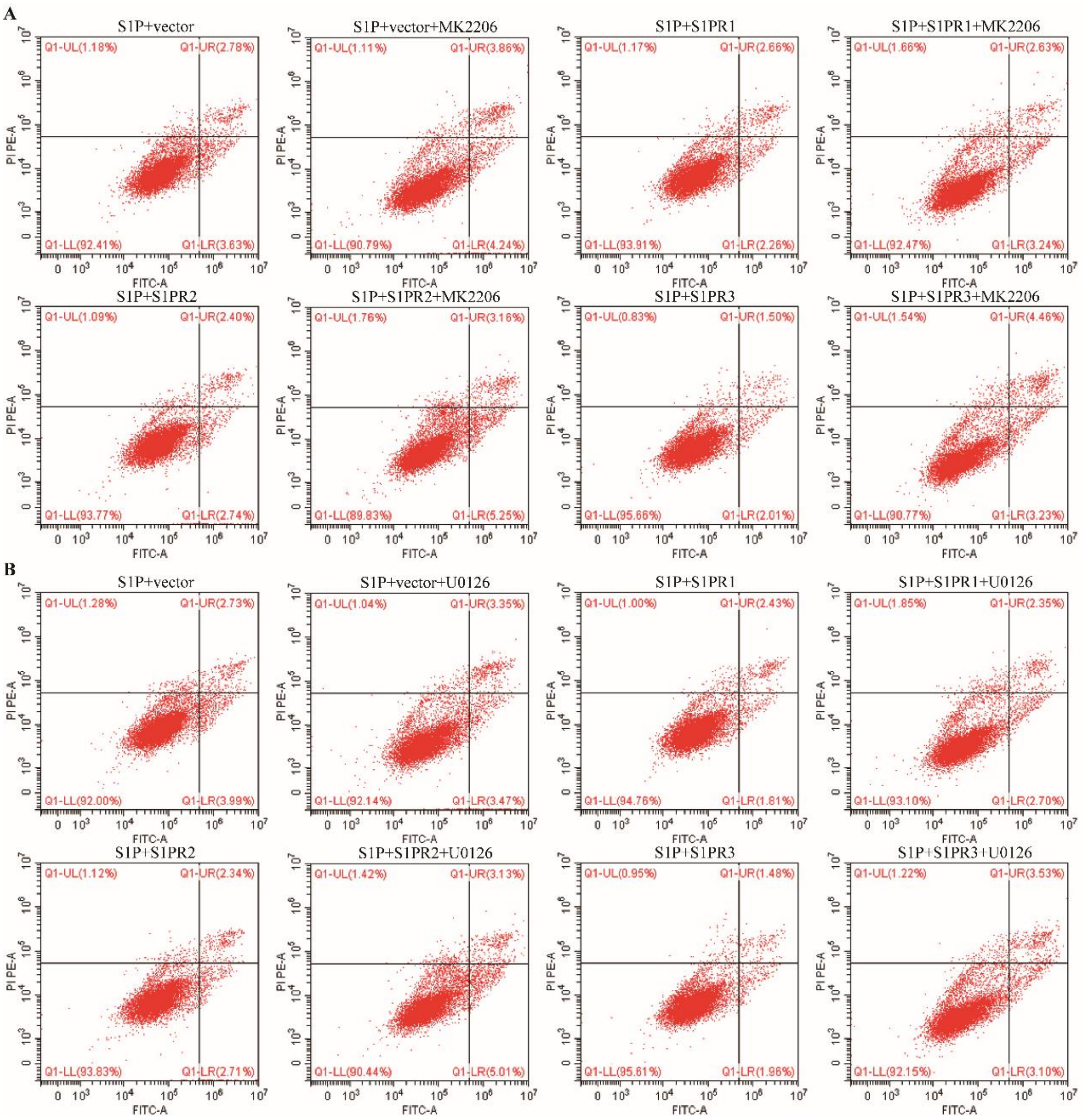

Fig S7. The effect of MK2206 and U0126 on apoptosis of WPMY-1 cells.

(A) The effect of MK2206 on apoptosis of WPMY-1 cells. **(B)** The effect of U0126 on apoptosis of WPMY-1 cells.

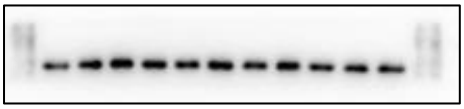

Figure 1A(vi) GAPDH

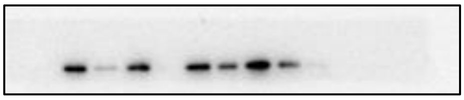

Figure 1A(vi) S1PR1

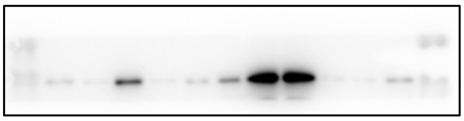

Figure 1A(vi) S1PR2

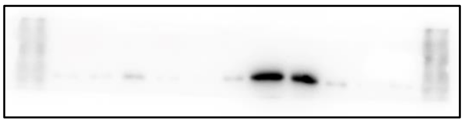

Figure 1A(vi) S1PR3

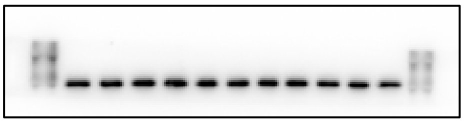

Figure 1B(vi) GAPDH

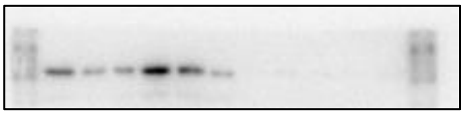

Figure 1B(vi) S1PR1

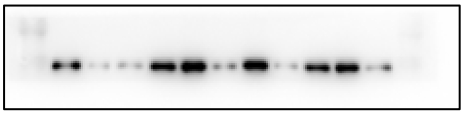

Figure 1B(vi) S1PR2

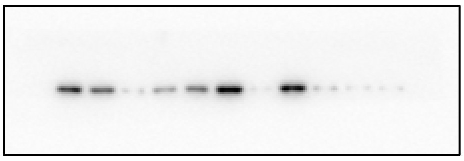

Figure 1B(vi) S1PR3

Data S1. All raw images of Western-blotting, related to Figure 1.

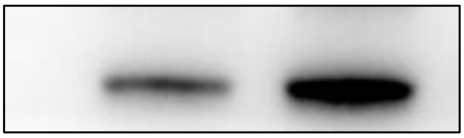

Figure 3F Cyclin D1 (left)

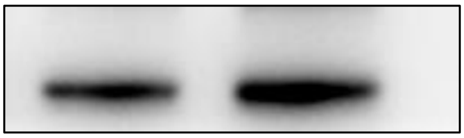

Figure 3F CDK4 (left)

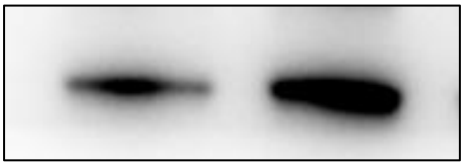

Figure 3F CDK6 (left)

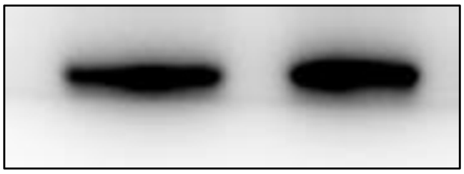

Figure 3F BCL2 (left)

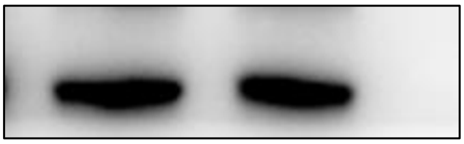

Figure 3F BAX (left)

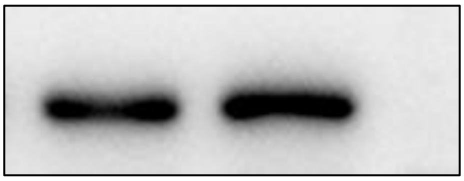

Figure 3F GAPDH (left)

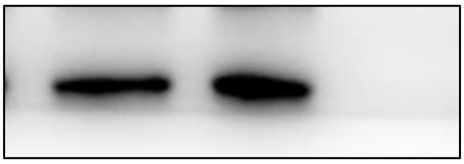

Figure 3F Cyclin D1 (right)

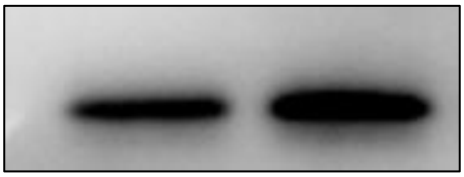

Figure 3F CDK4 (right)

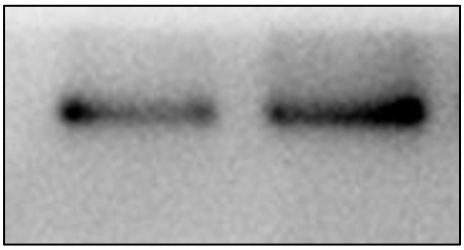

Figure 3F CDK6 (right)

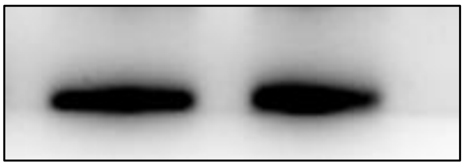

Figure 3F BCL2 (right)

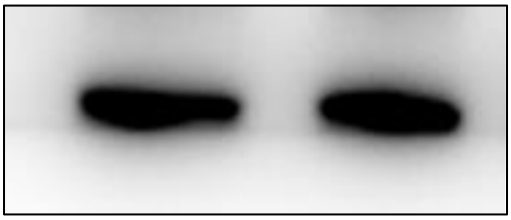

Figure 3F BAX (right)

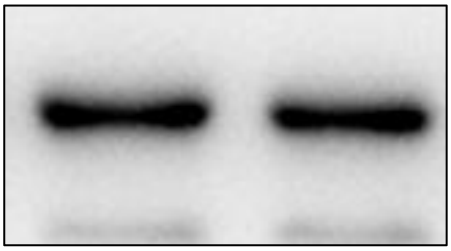

Figure 3F GAPDH (right)

Data S2. All raw images of Western-blotting, related to Figure 3.

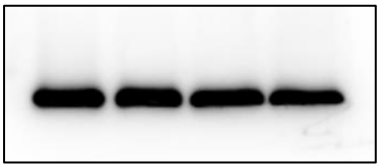

Figure 4A(i) GAPDH(S1PR1)

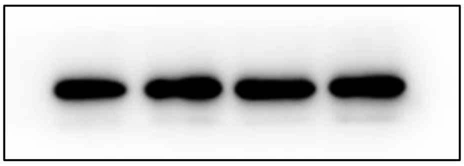

Figure 4A(i) GAPDH(S1PR2)

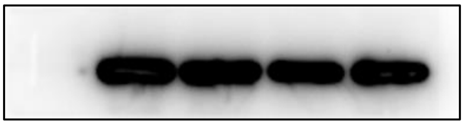

Figure 4A(i) GAPDH(S1PR3)

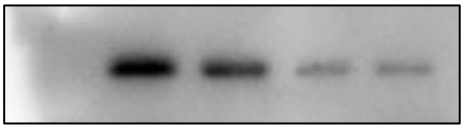

Figure 4A(i) S1PR1

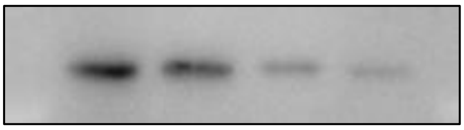

Figure 4A(i) S1PR2

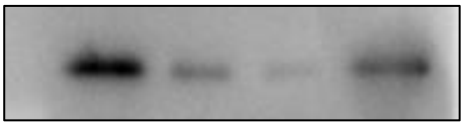

Figure 4A(i) S1PR3

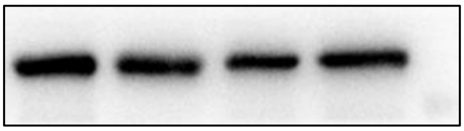

Figure 4A(ii) GAPDH(S1PR1)

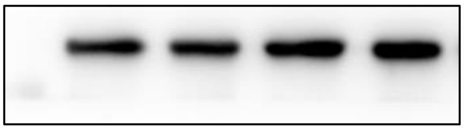

Figure 4A(ii) GAPDH(S1PR2)

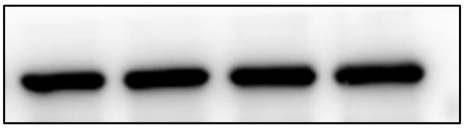

Figure 4A(ii) GAPDH(S1PR3)

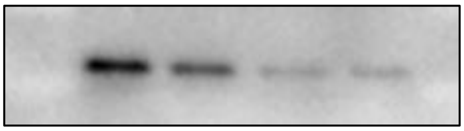

Figure 4A(ii) S1PR1

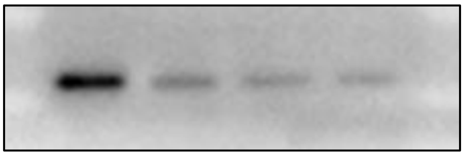

Figure 4A(ii) S1PR2

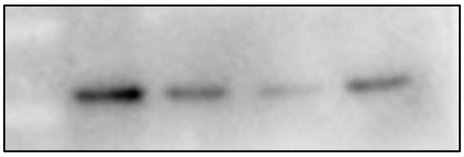

Figure 4A(ii) S1PR3

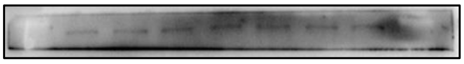

Figure 4D AKT(RWPE1)

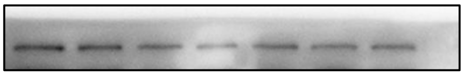

Figure 4D AKT(WPMY1)

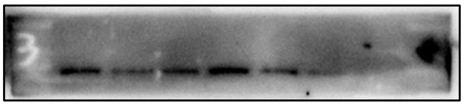

Figure 4D CDK4(RWPE1)

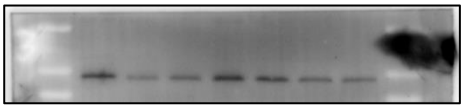

Figure 4D CDK4(WPMY1)

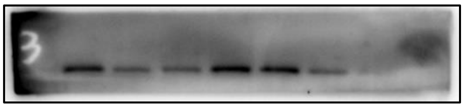

Figure 4D CDK6(RWPE1)

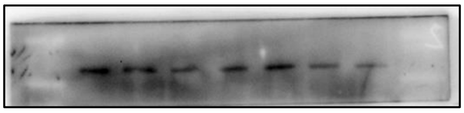

Figure 4D CDK6(WPMY1)

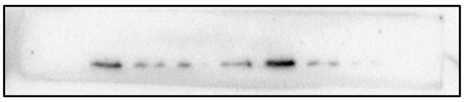

Figure 4D Cyclin D1(RWPE1)

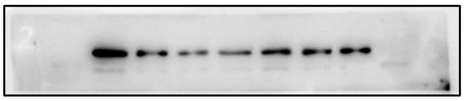

Figure 4D Cyclin D1(WPMY1)

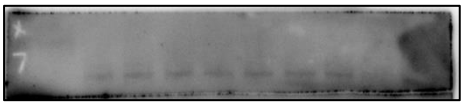

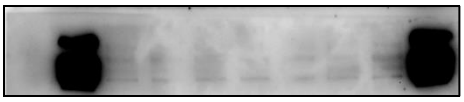

Figure 4D ERK(RWPE1)

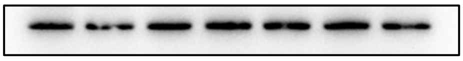

Figure 4D GAPDH(RWPE1)

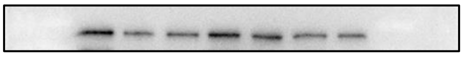

Figure 4D pAKT(RWPE1)

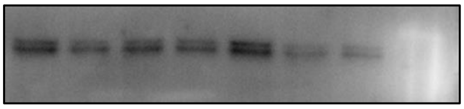

Figure 4D pERK(RWPE1)

Figure 4D ERK(WPMY1)

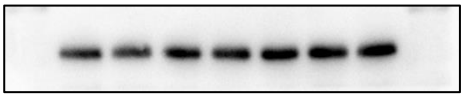

Figure 4D GAPDH (WPMY1)

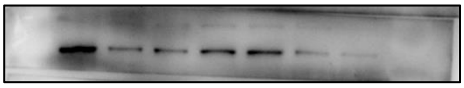

Figure 4D pAKT(WPMY1)

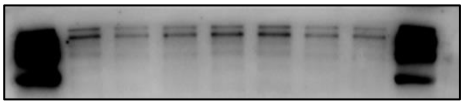

Figure 4D pERK(WPMY1)

Data S3. All raw images of Western-blotting, related to Figure 4.

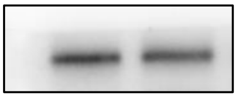

Figure 5A(i) GAPDH(S1PR1)

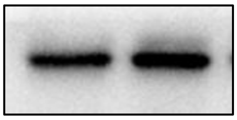

Figure 5A(i) GAPDH(S1PR2)

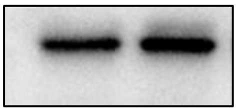

Figure 5A(i) GAPDH(S1PR3)

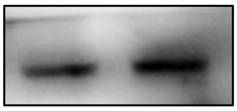

Figure 5A(i) S1PR1

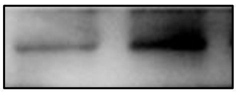

Figure 5A(i) S1PR2

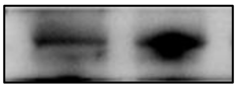

Figure 5A(i) S1PR3

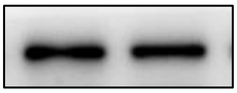

Figure 5A(ii) GAPDH(S1PR1)

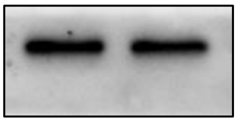

Figure 5A(ii) GAPDH(S1PR2)

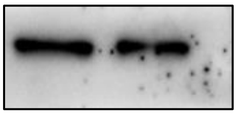

Figure 5A(ii) GAPDH(S1PR3)

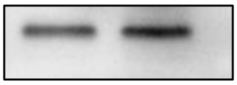

Figure 5A(ii) S1PR1

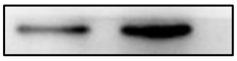

Figure 5A(ii) S1PR2

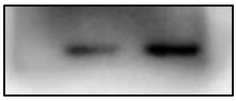

Figure 5A(ii) S1PR3

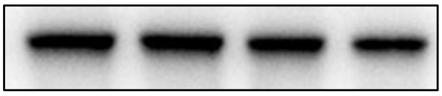

Figure 5D AKT(RWPE1)

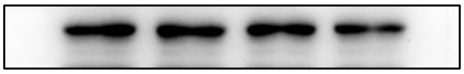

Figure 5D AKT(WPMY1)

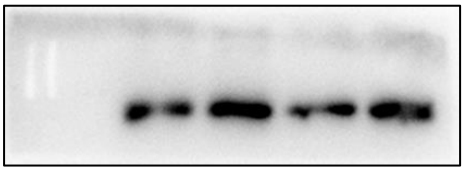

Figure 5D CDK4(RWPE1)

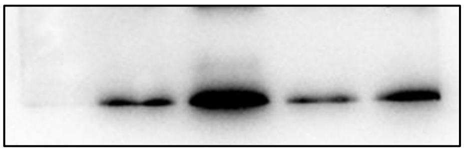

Figure 5D CDK4(WPMY1)

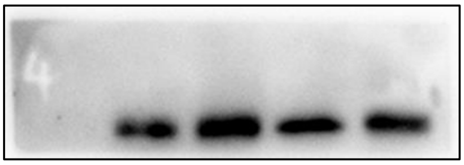

Figure 5D CDK6(RWPE1)

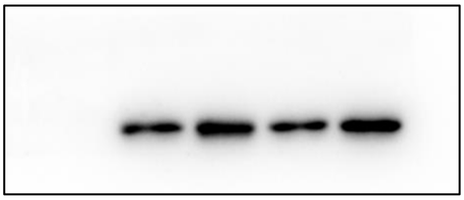

Figure 5D CDK6(WPMY1)

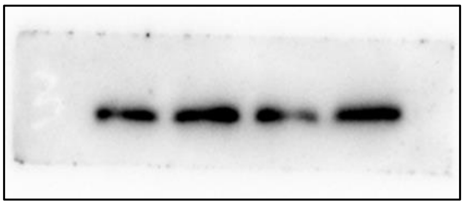

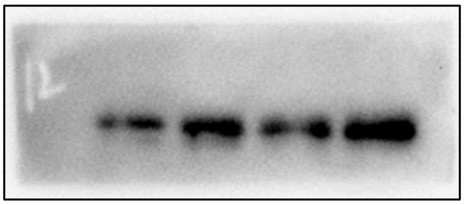

Figure 5D Cyclin D1(RWPE1)

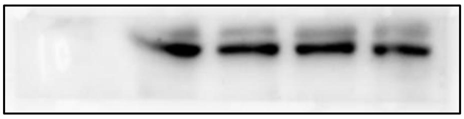

Figure 5D ERK(RWPE1)

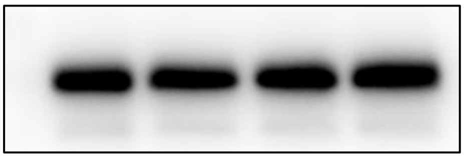

Figure 5D GAPDH(RWPE1)

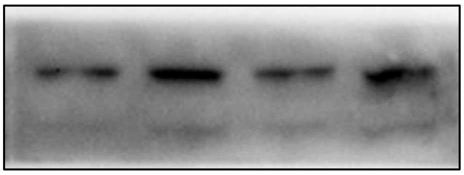

Figure 5D pAKT(RWPE1)

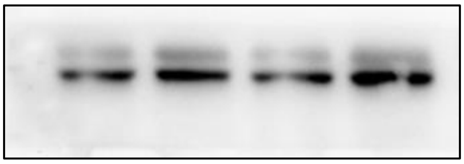

Figure 5D pERK(RWPE1)

Figure 5D Cyclin D1(WPMY1)

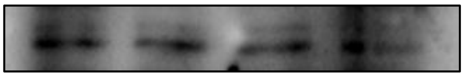

Figure 5D ERK(WPMY1)

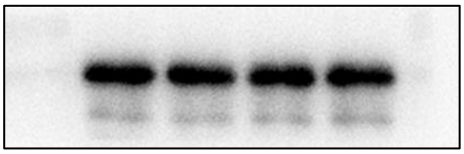

Figure 5D GAPDH(WPMY1)

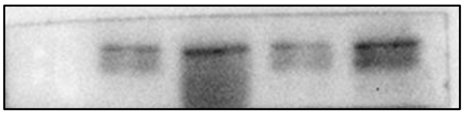

Figure 5D pAKT(WPMY1)

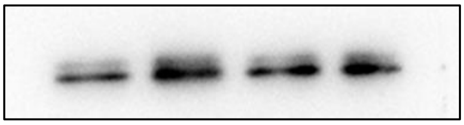

Figure 5D pERK(WPMY1)

Data S4. All raw images of Western-blotting, related to Figure 5.

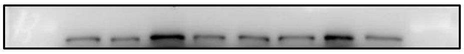

Figure 6B(i) AKT

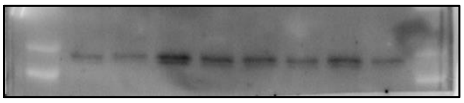

Figure 6B(i) CDK4

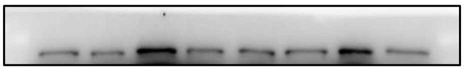

Figure 6B(i) CDK6

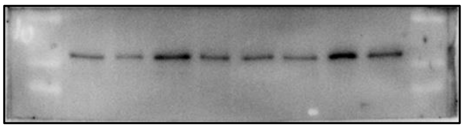

Figure 6B(i) Cyclin D1

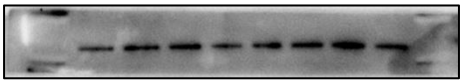

Figure 6B(i) GAPDH

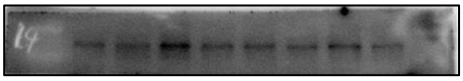

Figure 6B(i) pAKT

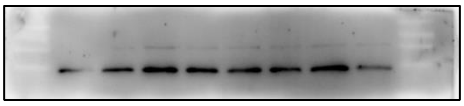

Figure 6B(ii) CDK4

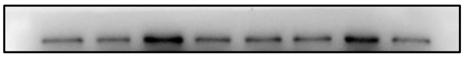

Figure 6B(ii) CDK6

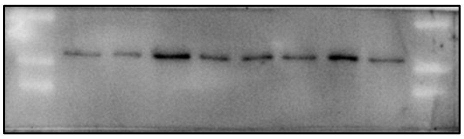

Figure 6B(ii) Cyclin D1

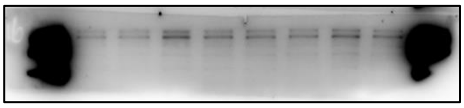

Figure 6B(ii) ERK

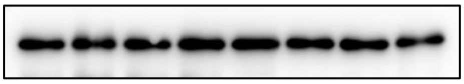

Figure 6B(ii) GAPDH

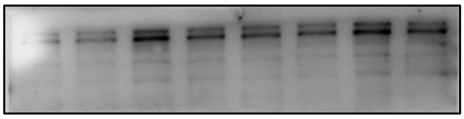

Figure 6B(ii) pERK

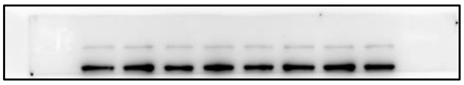

Figure 6D(i) AKT

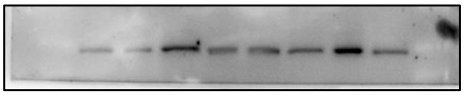

Figure 6D(i) CDK4

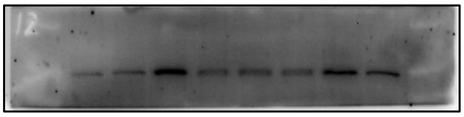

Figure 6D(i) CDK6

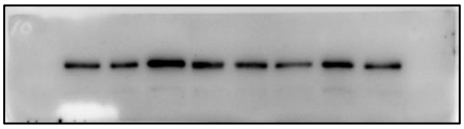

Figure 6D(i) Cyclin D1

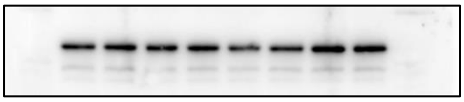

Figure 6D(i) GAPDH

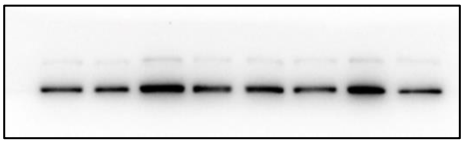

Figure 6D(i) pAKT

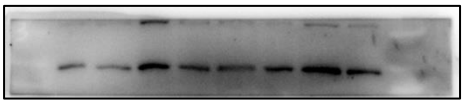

Figure 6D(ii) CDK4

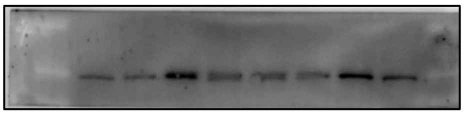

Figure 6D(ii) CDK6

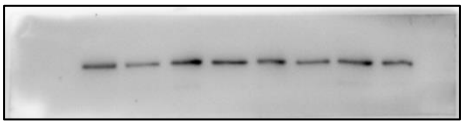

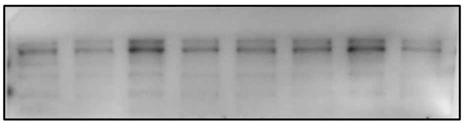

Figure 6D(ii) Cyclin D1

Figure 6D(ii) ERK

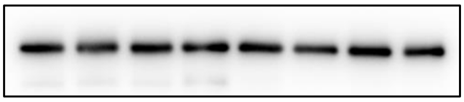

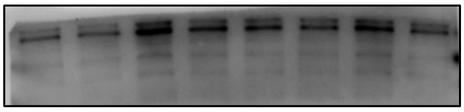

Figure 6D(ii) GAPDH

Figure 6D(ii) pERK

Data S5. All raw images of Western-blotting, related to Figure 6.

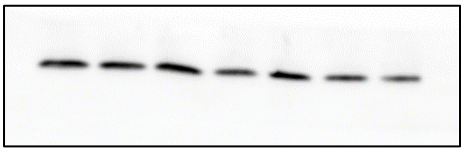

Figure 8C RhoA(left)

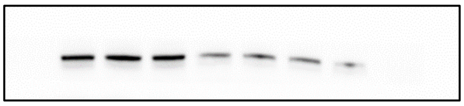

Figure 8C ROCK1(left)

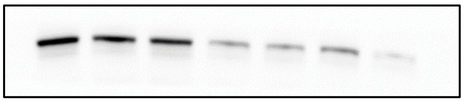

Figure 8C ROCK2(left)

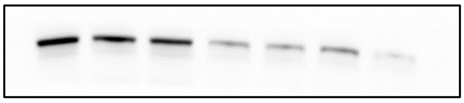

Figure 8C GAPDH(left)

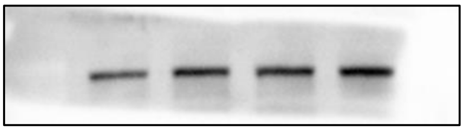

Figure 8C RhoA(right)

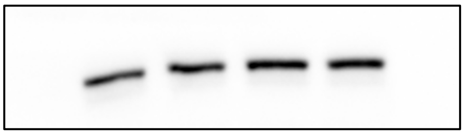

Figure 8C ROCK1(right)

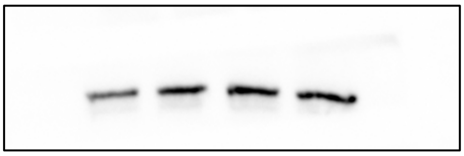

Figure 8C ROCK2(right)

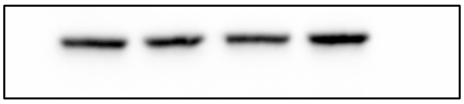

Figure 8C GAPDH(right)

Data S6. All raw images of Western-blotting, related to Figure 8.

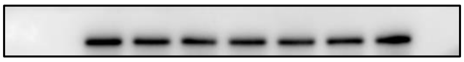

Figure 9E GAPDH(left)

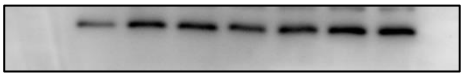

Figure 9E IL-6(left)

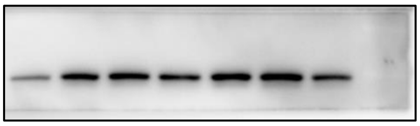

Figure 9E IL-8(left)

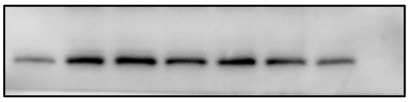

Figure 9E pSTAT3(left)

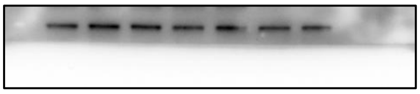

Figure 9E STAT3(left)

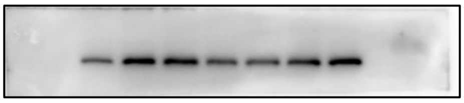

Figure 9E TNF-α(left)

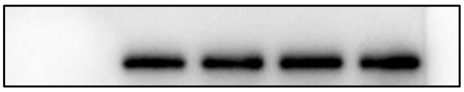

Figure 9E GAPDH(right)

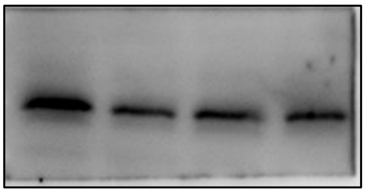

Figure 9E IL-6(right)

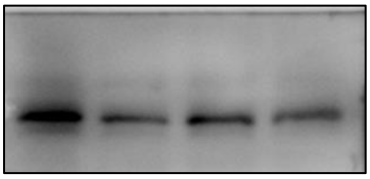

Figure 9E IL-8(right)

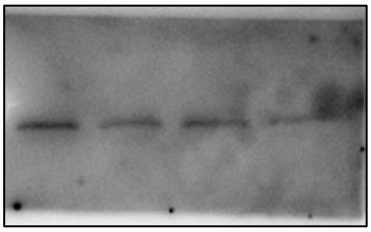

Figure 9E pSTAT3(right)

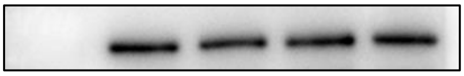

Figure 9E STAT3(right)

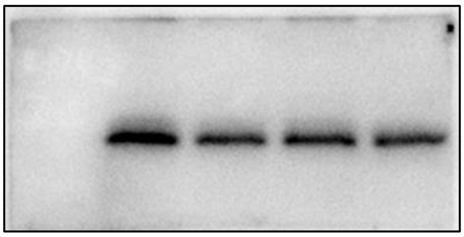

Figure 9E TNF-α(right)

Data S7. All raw images of Western-blotting, related to Figure 9.
